# The Years 2015–2025 as a Prospective Decade for the Identification of Specific Methylation Biomarkers of Prostate Cancer

**DOI:** 10.3390/biom15091334

**Published:** 2025-09-18

**Authors:** Zohair Selmani, Paul Peixoto, Alexis Overs, Eric Hervouet

**Affiliations:** 1 Department of Oncobiology, University Hospital of Besançon, 3 Boulevard Alexandre Fleming, F-25000 Besançon, France; zselmani@chu-besancon.fr (Z.S.); aovers@chu-besancon.fr (A.O.); 2 Université Marie et Louis Pasteur, EFS, INSERM UMR1098 RIGHT, F-25000 Besançon, France; paul.peixoto@univ-fcomte.fr

**Keywords:** prostate, cancer, DNA methylation, biomarker, diagnosis, liquid biopsies

## Abstract

For ten years, DNA methylation appeared as a major step in the understanding and issues of prostate cancers. Indeed, although classical biochemical parameters are still useful for prostate cancer diagnosis, they have poor sensitivity and are not specific for prostate cancer subtypes. The recent boom in the identification of specific DNA methylation profiles and the rapid development of liquid biopsies have completely modified the care of patients and may greatly influence outcomes in the future. Indeed, DNA methylation modifications could substantially improve the diagnosis by identifying specific prostate subtypes, improve follow-up to monitor residual disease, improve therapeutic efficiency by predicting the response to treatment, and improve the health quality of patients since these epigenetic modifications can easily be detected in non-invasive liquid biopsies.

## 1. State of the Art

Prostate cancer (PCa) remains a major global health concern, with approximately 1.6 million new cases diagnosed annually and an estimated 366,000 deaths worldwide. While the 5-year survival rate for localized PCa is relatively high (93%), recurrence and metastasis account for most PCa-related mortality. For metastatic prostate cancer (mPCa), the 5-year survival rate declines sharply to around 40%.

For decades, PCa diagnosis and patient stratification have relied primarily on biochemical and histological markers. However, current diagnostic tools, including prostate-specific antigen (PSA) testing, digital rectal examination, and tissue biopsies, suffer from limited sensitivity and specificity, often leading to over-diagnosis and over-treatment. This underscores the urgent need for robust and specific biomarkers capable of improving early detection, risk stratification for recurrence and metastasis, and prediction of treatment resistance. Ideally, such biomarkers should be (i) non-invasive, (ii) cost-effective, (iii) highly specific, and (iv) highly sensitive to ensure meaningful clinical applicability. DNA methylation, a well-characterized and mitotically heritable epigenetic modification, plays a fundamental role in the regulation of gene expression. This process involves the addition of a methyl group to the fifth carbon of cytosine residues in CpG dinucleotides, catalyzed by DNA methyltransferases (DNMTs). DNMT1 primarily maintains methylation patterns during DNA replication, while DNMT3A and DNMT3B mediate de novo methylation independently of DNA synthesis. De novo methylation tightly regulates gene expression during embryogenesis and is frequently dysregulated in cancer, contributing to widespread patterns of aberrant hypermethylation and hypomethylation—both now recognized as molecular hallmarks of malignancy [1].

**Figure 1:**
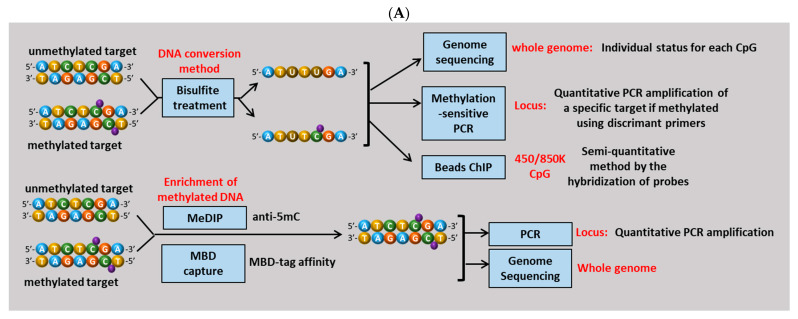

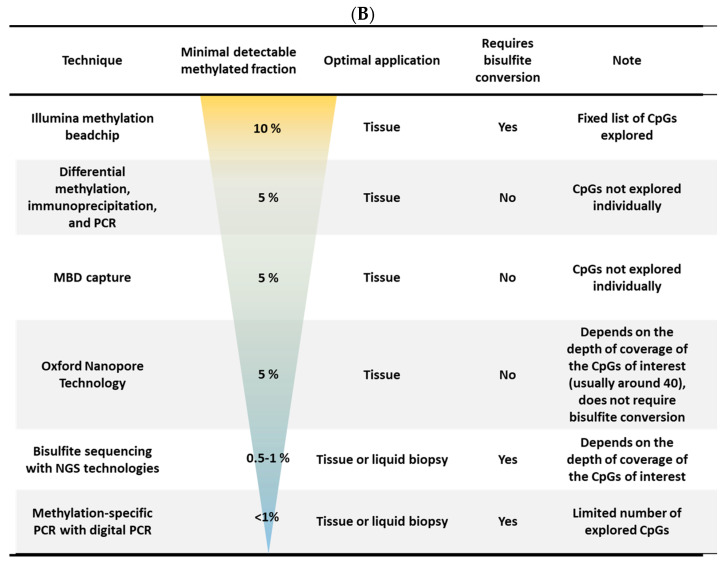
Experimental strategies for analyzing methylated DNA. (**A**) Principles of the different techniques used for quantifying DNA methylation. (**B**) Classification of these techniques in regard of performance.

Despite its reversible nature, DNA methylation is a remarkably stable and often displays patterns that are both tissue-specific and tumor-subtype-specific. Over the past decade, these features have fueled growing interest in methylation profiling as a source of clinically relevant biomarkers for PCa. Importantly, because methylation patterns can be influenced by ethnicity, it is unlikely that a single universal biomarker will be applicable across all populations. For this reason, whenever possible, the geographic or ethnic origin of the studied cohorts are reported in this review to provide the appropriate contextual interpretation. Different technical approaches performed by the authors to analyze DNA methylation are summarized in Figure 1.

## 2. Diagnosis

Over the past decade, numerous differentially methylated CpGs or genes (DMCpGs or DMGs) have been reported in PCa and proposed as potential diagnostic biomarkers. For example, *DEFB1* hypermethylation at two specific CpGs was identified in a Korean cohort (pyrosequencing; paired PCa/adjacent tissue samples, *n* = 60) [2]. In PCa cell lines (DU145 and PC-3), methylation showed an inverse correlation with *DEFB1* expression (RT-PCR). This was restored upon treatment with 5-azadC, strongly supporting promoter methylation-mediated regulation of *DEFB1*. Similarly, *CAMK2N1*, a well-established tumor suppressor gene (TSG) implicated in PCa, was found to be downregulated via promoter hypermethylation in a subset of PCa cell lines and in patient samples (both TCGA database and local cohort: pyrosequencing; BPH *n* = 16, PCa *n* = 52) compared to the normal prostate. 5-azadC treatment reduced *CAMK2N1* promoter methylation, and chromatin immunoprecipitation confirmed DNMT1 recruitment to this locus in untreated cells [3].

Conversely, the hypomethylation-associated upregulation of specific genes may also serve as diagnostic indicator for PCa. Increased expression of *FASN*, an androgen-regulated gene, was observed at both the mRNA and protein levels (IHC) in PCa tissues compared to the normal prostate. Analyses of publicly available datasets (H450K and BC platforms) demonstrated that *FASN* expression is regulated by DNA methylation in these tumors [4]. Likewise, the *TFF3* promoter was significantly hypomethylated in PCa relative to control tissues (*p* < 0.001) in a large Danish cohort (qMSP; PCa *n* = 292 and normal prostate *n* = 33) [5] and in TCGA data (PCa *n* = 497 and control tissues *n* = 50).

### 2.1. Diagnosis: Leveraging Public Whole-Genome DNA Methylation Profiles for Prostate Cancer Biomarker Discovery

Public databases contain large collections of methylome and transcriptome profiles spanning a wide range of cancers that are compiled from multiple independent studies. Leveraging these datasets can significantly enhance the robustness and reproducibility of biomarker discovery efforts. In this context, methylation data from both TCGA (Human 450K BeadChip; PCa, *n* = 451; adjacent non-tumor tissues, *n* = 50) and GEO (PCa, *n* = 219; control tissues, *n* = 169) were analyzed to re-evaluate the diagnostic performance of previously reported prostate cancer-associated differentially methylated genes (DMGs), including *APC*, *CCND2*, *GSTP1*, *PRKY*, and *RASSF1*. This re-analysis confirmed that all five genes displayed significantly higher methylation levels in prostate cancer samples compared to control samples [6]. Among the genes analyzed, *GSTP1* methylation demonstrated the highest diagnostic performance (AUC = 0.939), whereas *RASSF1A* methylation showed the lowest performance (AUC = 0.700). A combined methylation score based on *GSTP1* and *CCND2* yielded an AUC of 0.937, indicating improved diagnostic accuracy. For both genes, promoter methylation levels were inversely correlated with gene expression, consistent with their roles as TSGs. The molecular mechanisms underlying the aberrant methylation of *GSTP1* and *RASSF1A* have been previously characterized. In prostate cancer, the upregulation of REX1 has been shown to recruit DNMT3B to the *RASSF1A* promoter, leading to its transcriptional silencing via a de novo methylation-dependent mechanism [7]. For *GSTP1*, formation of the piR31470/PIWIL4 RNA complex, which binds nascent *GSTP1* RNA, facilitates DNMT3a recruitment and de novo methylation of the *GSTP1* promoter [8].

Public databases can also be queried in an unbiased fashion to identify novel and robust putative biomarkers, without restricting analyses to predefined candidate genes. Depending on the research objective, such datasets can be used to identify the most frequent differentially methylated CpG sites (DMCpGs) or genes (DMGs), those with the largest methylation differences, or those with the highest statistical significance, specificity, or sensitivity. This approach has been widely applied in prostate cancer biomarker research. For example, the integration of methylome and transcriptome data from the GEO database (microarray datasets: PCa, *n* = 28; normal prostate, *n* = 23; H450K BeadChip: PCa, *n* = 73; control tissues, *n* = 63) led to the identification of 105 hypomethylated genes with concomitantly increased expression and 561 hypermethylated genes with reduced expression in PCa tissues compared to control tissues [9]. Using TCGA methylome data (PCa *n* = 423 and adjacent tissues *n* = 39), 1585 DMCpGs within promoters were identified [10] and a panel of 8 DMCpGs across six promoters (*CBX5*, *CCDC8*, *CYBA*, *EFEMP1*, *KCNH2*, and *SOSTDC1*) were selected by PAM analysis. These DMGs were confirmed in the GEO database, and except for *CBX5*, TCGA transcriptome data revealed a strong inverse correlation between methylation and gene expression. Individual AUCs were all ≥0.91 for cancer classification, suggesting these DMGs may serve as strong candidate biomarkers for PCa diagnosis. Using TCGA methylome data (PCa *n* = 400 and normal tissues *n* = 49) together with expression profiles, 1285 differentially expressed genes (DEGs) associated with DMCpGs were identified [11]. Cross-validation with external cohorts (GEOcancer, *n* = 397; METcancer, *n* = 397; and METnormal, *n* = 49) yielded a refined list of 100 DMGs. Subsequent LASSO regression identified a diagnostic panel of seven DMGs (hypermethylated—*CCK*, *CD38*, *CYP27A1*, *EID3*, *LRRC4*, *LY6G6D*; and hypomethylated—*HABP2*).

To identify hub genes as methylation biomarkers, Xu et al. (2019) integrated PCa methylome data from TCGA (*n* = 500) with GEO (PCa *n* = 80 and control tissues *n* = 79) with a protein–protein interaction network [12], identifying six differentially methylated driver genes (*AKR1B1*, *COL4A6*, *MAOB*, *GPX3*, *GSTM2*, and *RTP4*). Using TCGA methylomes (PCa *n* = 469, control tissues *n* = 50) and a deep learning approach, ref. [13] Nikas & Nikas (2019) also identified a set of five DMCpGs (three within the genes *LINC01091*, *RPS15*, and *SNORA10*) that distinguished PCa from control tissues with 95% sensitivity, 94% specificity, and an AUC of 0.9 in both training and validation sets. Similarly, a comparison of GEO methylomes (HM450K; PCa *n* = 175 and control tissues *n* = 87) and transcriptomes (PCa *n* = 140 and control tissues *n* = 53) revealed 103 DEGs inversely associated with the methylation status [14]. Nine were well-known TSGs, six of which (*ALPL*, *DAPK1*, *FBP1*, *PPM1A*, *PYHIN1* and *SMCHD1*) were validated in TCGA.

Because methylation data from PCa tissues can be confounded by the immune cell content, Reynolds et al. (2024) analyzed TCGA methylation and expression data from 56 paired samples, adjusting for micro-tumoral deconvolution (Epigenome-Wide Association Study: EWAS) to reduce background noise [15]. They identified 2093 DMCpGs mapped in 717 genes. The five most altered DMGs (by CpG count) were *CCD181*, *CPVL*, *GPR84-AS1*, *ALK*, and *LINC01929*. After adjusting for age, race, Gleason score, and PSA level, a final set of 51 DMGs remained that were theoretically independent of immune-related confounding.

### 2.2. Diagnosis: Mining Public Whole-Genome Methylomes for Pan-Cancer Biomarkers Applicable to Prostate Cancer Diagnosis

In addition to the development of PCa-specific biomarkers, research efforts have been directed toward identifying pan-cancer biomarkers suitable for large-scale clinical screening. Such biomarker panels could incorporate cancer-type-specific markers (e.g., PCa-associated markers) to further refine diagnoses when necessary. Liu et al. (2019) [16] employed a deep learning-based approach integrated with LASSO regression on a dataset composed of 10,140 methylomes (Illumina 450K BeadChip) across 27 distinct cancer subtypes, alongside 3386 methylomes from various healthy tissues. The study identified two distinct biomarker panels: one based on differentially methylated CpG sites (DMCpGs), and another based on promoter methylation profiles (differentially methylated genes, DMGs). Both panels demonstrated high accuracy in cancer detection across multiple types. Specifically, for PCa, the models achieved sensitivities ranging from 85% to 96%, with AUC values of 0.96 and 0.92 in the training and validation cohorts, respectively.

Neefs et al. (2025) [17] recently proposed a multiplex droplet digital PCR (ddPCR) panel capable of detecting eight different cancers, including PCa. To develop this panel, they first identified 1792 DMGs using TCGA and retained 27 of the top 40 targets after applying a primer design filter. A subset of three DMGs (*Chr5q14.1*, *EMX1*, and *NXPH1*), which showed the highest selectivity between control blood samples and cancer cell lines in ddPCR, was tested in different cancer tissues paired with adjacent normal tissues (frozen samples: PCa *n* = 14 vs. adjacent *n* = 24; lung cancer *n* = 20 vs. adjacent *n* = 22, invasive breast cancer *n* = 10 vs. adjacent *n* = 7, colorectal cancer *n* = 7 vs. 10, pancreatic carcinoma *n* = 17 vs. 23, hepatocarcinoma *n* = 12 vs. 10, squamous cell esophagus carcinoma *n* = 12 vs. 5, squamous cell head and neck carcinoma *n* = 13 vs. 8, and healthy donor blood *n* = 20). In PCa, the sensitivity and specificity for cancer detection were 100% and 91.7% for *EMX1*, 78.6% and 91.7% for *Chr5q14.1*, 57.1%, and 91.7% for *NXPH1*, and reached 100% and 87.5% for the combined triplex methylation score.

### 2.3. Diagnosis: Whole-Genome Methylomes from Independent Cohorts for the Identification of New Diagnostic Biomarkers Useful for PCa

To avoid possible bias arising from public databases (e.g., normalization, lack of specific information, specific populations, and technical limitations) some studies have used independent patient cohorts to identify more specific diagnosis-related biomarkers. For example, a comparison of methylomes (H450K BC) between PCa and adjacent tissues (*n* = 20) identified 2040 DMCpGs, of which the top 27 were hypermethylated and subsequently confirmed in TCGA database. Three out of twenty-seven were located within the *AOX1*, *RHCG*, and *TMEM106A* genes and exhibited high methylation coverage in their promoters [18]. Kim et al. (2023) [19] also identified 1251 DMCpGs in a Korean cohort (enzymatic conversion pyrosequencing; PCa *n* = 42, BPH *n* = 11). This signature was highly discriminant in separating benign tissues and PCa (AUC = 0.98 for hypomethylated DMCpGs and AUC = 0.99 for hypermethylated DMCpGs) [19].

### 2.4. Diagnosis: Performance Tests of Biomarker Sets for PCa Diagnosis in Tissue Biopsies/Tumors

A few DMGs (such as *APC*, *GSTP1*, and *RASSF1A*), which were among the first candidates proposed for PCa diagnosis, have been extensively investigated. An Italian FFPE-based cohort (qMSP and IHC; PCa/adjacent-paired *n* = 56) confirmed that *GSTP1* methylation was significantly more frequent in PCa (51/56) than in control tissues (3/56) and that *GSTP1* expression appeared to be regulated by promoter methylation (*p* < 0.001) [20]. Similarly, *GSTP1* (*p* < 0.01) methylation, but not *RASSF1A* methylation, was also significantly associated with PCa in a Vietnamese cohort (pyrosequencing; PC *n* = 59, and PBH *n* = 37) [21].

A multicentric study (Canada, Japan, UK) evaluated 15 previously identified DMGs for their ability to detect early PCa using three tissue cohorts (qMSP; early PCa *n* = 699) [22]. Seven out of fifteen DMGs (*APC*, *CCDC181*, *GAS6*, *GSTM2*, *GSTP1*, *HAPLN3*, and *RASSF1A*) individually showed a significant separation between early PCa and benign tissues. Notably, a combined methylation score using only three of these targets (*GAS6*, *GSTP1*, and *HAPLN3*) achieved a sensitivity of 94% and a specificity of 93% (AUC = 0.97). To identify optimal biomarkers from a list of 40 DMGs previously described with diagnostic potential, Gurioli et al. (2016) [23] performed methylation-sensitive multiplex ligation-dependent probe amplification (MS-MPLA) in an Italian cohort (PCa *n* = 40, adjacent tissues *n* = 26, normal prostates *n* = 23). The methylation of *CASP8* and *SCGB3A1* was significantly different between control and adjacent tissues, suggesting these DMGs may also serve as early biomarkers. Overall, 12 of the 40 DMGs significantly distinguished tumors from normal prostates. A validation cohort (qMSP; PCa/adjacent paired tissues *n* = 40) confirmed 9/12 DMGs, but only 5 (*CCND2*, *RARB*, *RASSF1*, *SCGB3A1*, and *GSTP1)* displayed high statistical significance (AUC = 0.89–0.95). Another study analyzed the methylation status of several additional candidates in clinically negative PCa biopsies from an Italian cohort (pyrosequencing; negative biopsies *n* = 111 and control tissues *n* = 129) [5]. The results indicated that the detection of *GSP1* methylation in negative biopsies could predict undetected PCa cases (OR = 1.14 per 1% methylation increase).

An American multicenter study [24] also evaluated the methylation of six previously identified PCa-related DMGs (pyrosequencing; PCa *n* = 52, normal tissues *n* = 77), confirming the hypermethylation of *CAV1*, *EVX1*, *PLAG2G16*, and *SPAG4* and hypomethylation of *FGF1* and *NCR2* in PCa relative to control tissues. Among these, specific DMCpGs within *EVX1* achieved the best individual performance (AUC = 0.61; *p* < 0.05). While the baseline PSA level alone yielded an AUC of 0.631, combining it with a methylation-score increased the AUC to 0.815 (*p* < 0.001). Interestingly, the analysis of the DMGs in samples from serial tissue biopsies showed a highly consistent methylation pattern between different biopsies from control patients. However, the correlation was lower in serial biopsies from individual PCa patients, confirming that tumor heterogeneity is an important factor in biomarker design. This last observation supports the use of a multi-marker panel—even when individual markers have similar performances—rather than relying on a single DMG biomarker.

### 2.5. Diagnosis: Performance Tests of Biomarker Sets for PCa Diagnosis in Liquid Biopsies

**Figure 2:**
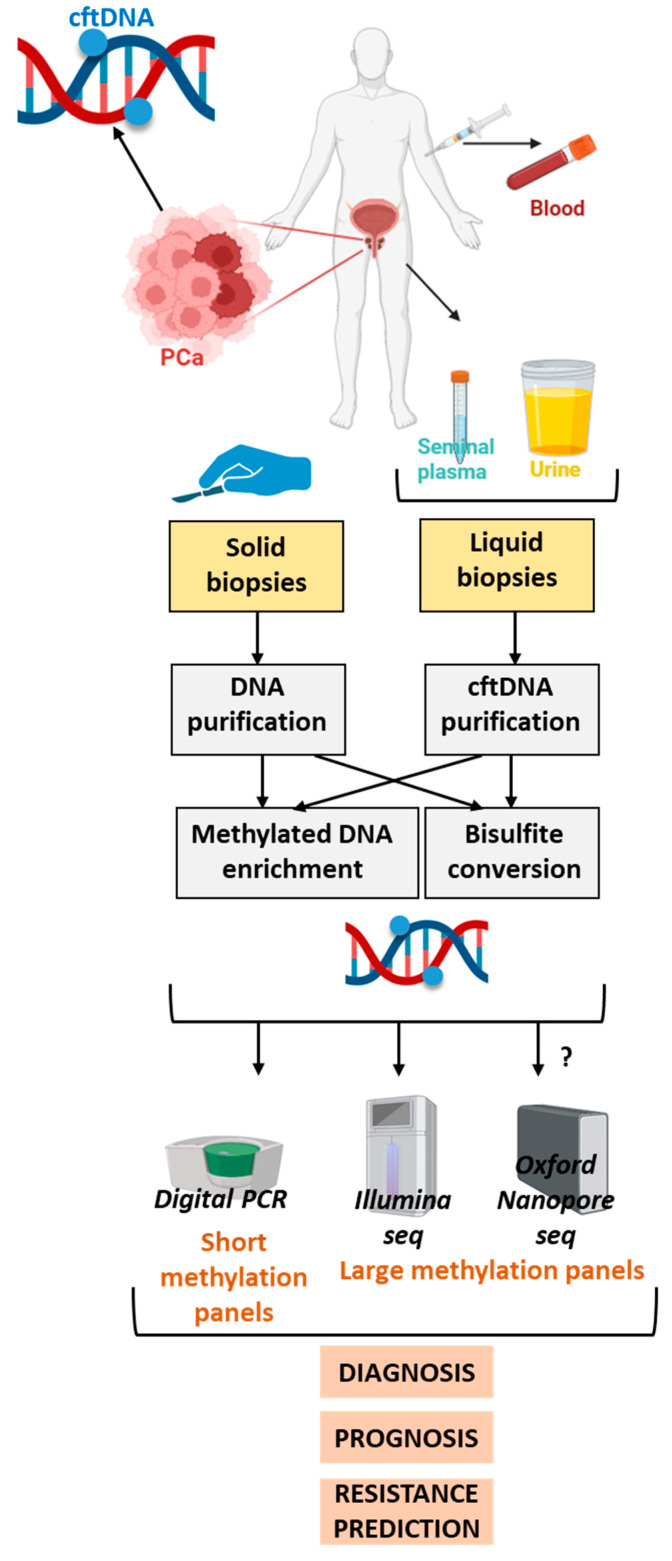
Identification and clinical application of DMRs in tissue and liquid biopsies. (Figure made using Biorender).

In recent years, it has become evident that the identification of DMGs could be applicable not only to solid tissue biopsies but also offer significant potential for reliable, non-invasive cancer diagnosis. Indeed, since tumors continuously release tumoral DNA, referred to as cell-free tumor DNA (cftDNA), it is now possible to detect specific DNA methylation patterns at a distance from the primary tumor using physiologic liquid samples (Figure 2). However, biomarkers that demonstrate high diagnostic performance in tissue samples may exhibit substantially reduced accuracy in liquid biopsies. This highlights the necessity for rigorous validation procedures before biomarkers are translated from tissue-based to liquid biopsy-based applications.

#### 2.5.1. Diagnosis: Performance Evaluation of Biomarker Panels for PCa Diagnosis in Blood

A panel of eight previously identified DMGs (*CDH13*, *CYB5R2*, *DRD2*, *HIN1*, *LINE-1*, *RARB2*, *SPARC*, and *TIMP3)* [25] was tested in blood samples collected from AA men (pyrosequencing; PCa patients *n* = 91 and HDs *n* = 91) [26]. Four genes (*CDH13*, *CYB5R2*, *RARB2* and *SPARC*) showed the expected significant hypermethylation (*p* < 0.05) in cancer patients compared with HDs, whereas *DRD2* and *LINE-1* were hypomethylated (*p* < 0.05), as anticipated. The AUC values for these markers ranged from 0.6 to 0.8, suggesting their potential utility for PCa diagnosis in plasma. However, the diagnostic performance of *LINE-1* hypomethylation measured by pyrosequencing was challenged in another study involving plasma from a large cohort (PCa patients *n* = 694; controls *n* = 703) [27]. It is worth noting that pyrosequencing exhibits limited sensibility when applied to liquid biopsies. Aykanli et al. (2024) [28] evaluated a three DMG panel (qMSP; *GSTP1*, *RASSF1*, and *RASSF2*) in the blood (PCa *n* = 13; high-grade intraepithelial neoplasia *n* = 3; BPH *n* = 20; atypical small acinar proliferation *n* = 3; HD *n* = 15). *RASSF2* achieved the highest individual performance, but the results were suboptimal (sensitivity of 69% and specificity of 39%) [28]. The specificity of the combined three DMG panel increased to 83%, although the sensitivity dropped to 8%.

Another three DMG panel (*GSTP1*, *KIAA1539*, and *RNF219*) assessed in a plasma collection (MSP coupled with bisulfite sequencing; PCa patients *n* = 20; BPH patients *n* = 17; HDs *n* = 18) showed that some CpGs within *GSTP1* presented the highest performance for distinguishing PCa patients using plasma [29]. Pyrosequencing of *GADD45A* in an American serum cohort (PCa patients *n* = 34 and patients with benign lesions *n* = 48) also confirmed significantly higher methylation in cancer patients compared to those with benign cases (*p* < 0.01) [30]. Using TCGA database (H450K BC; PCa *n* = 497; controls *n* = 50; blood cells from HDs *n* = 421) Friedemann et al. (2024) [31] identified *NRIP3* methylation as a new promising biomarker. When combined with previously described targets in a four DMG panel (*RASSF1A1*, *miR129-2*, *NRIP3*, and *SOX8*) [31] and applied to cfDNA from the blood of a German cohort (PCa *n* = 39; BPH *n* = 40; HD *n* = 90), a risk score integrating methylation data, the cfDNA concentration, patient age, and PSA level improved the specificity for detecting hyperplasia and reduced the over-diagnosis of indolent PCa.

Analyses of both GEO and TCGA databases showed that *PRKY* gene expression is downregulated in PCa via DNA methylation compared to controls [32]. This methylation was unrelated to clinical parameters (stage, metastatic status, or Gleason score) and may occur early in carcinogenesis. Although identifying cancer-subtype biomarker is challenging, UALCAN analysis suggested that the methylation of the *PRKY* promoter is absent in many other cancers (lung, liver, colorectal, esophageal, bladder, and pancreatic cancers). The detection of *PRKY* methylation by bisulfite sequencing (*n* = 19) in cfDNA from PCa patients was significantly higher in the clinically significant PCa patients compared to others [33]. Combining the methylation score with imagery yielded an AUC of 0.86.

In a Croatian cohort, bisulfite sequencing of nine CpGs in the *CAV1* gene (PCa *n* = 29 and BPH *n* = 40) [34] showed higher methylation levels in PCa compared to both BHP and control tissues (*p* = 0.04). However, it failed to discriminate BHP from PCa when tested in cfDNA (PCa patients *n* = 40; BPH patients *n* = 40). To address the challenge of translating effective tissue biomarkers into clinically useful cfDNA markers, Lleshi et al. (2024) identified methylation signatures directly from cfDNA [35]. Using PC-3 cell culture supernatant, cfMeDIP-seq and cfMBD-seq techniques were compared for their ability to detect cfDNA methylation. Both techniques, which enriched methylated DNA (via an anti-5mC antibody (MeDIP) or capture of methyl binding domain (MBD)), produced comparable results: from a list of 6285 methylated DNA fragments identified in PCa tissues, cfMeDIP-seq detected 80% and cfMBD-seq detected 88%. Subsequently, using the cfMBD-seq method (PCa patients *n* = 43; HDs *n* = 24) combined with machine learning, they identified a large panel of 900 DMCpGs for PCa diagnosis in cfDNA. This panel was validated in plasma samples from an independent cohort (localized PCa =19; HD *n* = 11) with an AUC of 0.96, supporting that cfMBD-seq is a highly accurate technique for the diagnosis of early PCa. These results suggest that the epigenetic profiles of PCa cell lines are representative of patients’ tumors.

#### 2.5.2. Diagnosis: Performance Tests of Biomarker Sets for PCa Diagnosis in Seminal Plasma

*LGALS3* promoter methylation has been proposed as a potential biomarker for cfDNA PCa diagnosis. Indeed, most of the seven CpGs within the *LGALS3* promoter exhibited lower methylation in hyperplasia samples compared with PCa samples (*n* = 27), and even lower levels in non-tumoral tissues (*n* = 21) (pyrosequencing) [35]. However, the overall methylation rate was relatively low (about 5%). Data obtained from cfDNA in blood samples or seminal plasma of both PCa and hyperplasia patients did not show significant differences, and the informative DMCpG obtained an AUC of only 0.66 in seminal plasma. Although the use of seminal plasma as a liquid biopsy source has been less extensively studied, it appears to be a promising and relevant medium for PCa detection.

##### Diagnosis: Performance Tests of Biomarker Sets for PCa Diagnosis in Urine

Urine is a highly accessible liquid biopsy and could represent a particularly valuable source for urothelial cancer diagnosis. The feasibility of detecting *GSTP1* promoter methylation in urine was first investigated in 2016. In a local cohort (PCa patients *n* = 31; BPH patients *n* = 34), *GSTP1* methylation was observed in 87% of prostate cancer samples compared to only 12% of BPH samples [36]. One of the first large-scale studies assessing urine as a source of methylation biomarkers for PCa diagnosis was published in 2018 [37]. This multicenter study (UK, Canada, and Ireland) analyzed the methylation status of previously identified DMGs (*APC*, *GSTP1*, *HOXD3*, *KLK10*, *TBX15*, and *TGFB2*) in urine collected from PCa patients (PCa *n* = 408 and BPH *n* = 182). In contrast with earlier findings, *GSTP1* methylation was among the least frequent events (39%), while *HOXD3* methylation was observed in 93% of cases. As discussed further (conclusions), this result might indicate that cftDNA is not fully representative of solid tumors. However, LASSO regression revealed an optimal combined *GSTP1*-*HOXD3* methylation score (sensitivity of 57%; specificity of 97%; AUC = 0.8) that was also able to discriminate between low-risk and high-risk tumors.

Two different methylation panels were subsequently evaluated using qMSP: panel #1 (singleplex *miR34c* and *miR193b*) and panel #2 (multiplex *APC*, *GSTP1*, and *RARB2*). These were tested in two Portuguese cohorts: tissue cohort 1 (PCa *n* = 74; normal prostate *n* = 16) and urine cohort 2 (PCa patients *n* = 87; HDs *n* = 32) [38]. In tissues, panel #1 achieved a sensitivity of 97% and specificity of 80% while panel #2 reached 100% for both. Comparable performances were obtained in the urine cohort, except for *GSTP1*, but the best results were obtained with *miR34c* and *miR193b* methylation (sensitivity of 95%; specificity of 92%; AUC = 0.88), confirming that *GSTP1* methylation is not the best target in urine. The combination of both panels yielded a sensitivity of 100% (AUC = 0.98). Both panels correlated with the Gleason score in tissues, while *APC* methylation was further associated with the recurrence risk, although this association was lost in urine. A prior H450K analysis of Portuguese tissue cohorts (frozen PCa *n* = 25; controls *n* = 5) identified DMCpGs in *miRNA* promoters. Six DMmiRNAs (*miR34b/c*, *miR129-2*, *miR152*, *miR193b*, *miR663a*, *miR1258*) were subsequently validated in a larger cohort (PCa *n* = 180; control tissues *n* = 15) by pyrosequencing, all with *p* < 0.01 and individual diagnostic AUCs ranging from 0.89 to 0.99. Among them, *miR1258* methylation displayed the highest accuracy (sensitivity 98%; specificity 100%) [39]. Evaluation of a urine cohort (PCa patients *n* = 95; non-urological cancer patients *n* = 29; HDs *n* = 17) confirmed significantly higher methylation levels of *miR34b/c*, *miR193b*, and *miR1258* in PCa patients, with *miR193b* showing the best performance (sensitivity = 92%; specificity = 96%; AUC = 0.96). Increased *miR193b* methylation in PCa vs. benign lesions was also independently validated in another study (MeDIPseq; CRPC *n* = 9; PCa *n* = 25; BPH *n* = 10; *p* < 0.0001) [40].

A panel of 19 DMCpGs (in 18 different DMGs) was next tested in urine samples from American men (PCa patients *n* = 42; controls *n* = 50) [41]. Individual markers showed specificities ranging from 57 (e.g., *HOXB5*) to 100% (e.g., CXCL14) and sensitivities from 13 to 97%. ROC analysis indicated that combining six of them considerably improved performance (sensitivity around 90%; specificity of 70%), while adding a seventh had only a minor impact.

Methylome profiling of PCa tissues (bisulfite sequencing; PCa *n* = 18; HD = 18) identified 4750 DMRs [42]. From the top 120 candidates tested in qMSP, 72 were validated (PCa *n* = 50; controls *n* = 35), leading to a panel of 14/72 DMGs (including *AKR1B1HES5*, *CHST11*, *GAS6*, *GRASP*, *ITPRIPL1*, *KCNB2*, *MAX.chr3.6187*, *AX.chr3.8028*, *SCOL3A1*, *SERPINB9*, *ST6GALNAC2*, *WNT3A*, and *ZNF655*) after multiplexing and filtering for its use in liquid biopsy. In urine (PCa *n* = 24; HD *n* = 24), this panel reached 100% specificity but only 59% sensitivity for discriminating PCa from control tissues. Khemees et al. (2021) [43] evaluated another signature (*CAV1*, *EVX1*, *FGF1*, *NRC2*, and *PLA2G16*) in urine from men with elevated PSA levels (pyrosequencing; *n* = 167). All but *NRC2* showed significantly higher methylation in PCa patients than HDs (*p* < 0.01). A multivariate model combining *EVX1* methylation and *PLA2G16* CpGs achieved an AUC of 0.77, outperforming PSA alone (AUC = 0.61). Similarly, *PRKY* methylation, which was previously validated in plasma and tissues (H450K BC; PCa/adjacent paired tissues *n* = 66) [32], was tested in urine (pyrosequencing; *n* = 135) [44]. *PRKY* methylation outperformed PSA baseline levels (AUC = 0.92 vs. 0.77), further supporting its clinical utility.

Finally, methylation-based panels have the potential to support pan-cancer diagnosis. In this context, a Portuguese cohort (plasma samples *n* = 133; including PCa *n* = 121) was tested with an eight-gene qMSP panel (*APC*, *FOXA1*, *GSTP1*, *HOXD3*, *RARB2*, *RASSF1A*, *SEPT9*, and *SOX17*) [45]. Several markers (*FOXA1* and *RASSF1A*) were methylated in both lung cancer and PCa, while *SEPT9* and *SOX17* were hypermethylated across lung, colorectal, and prostate cancers. In contrast, *GSTP1* hypermethylation was largely specific to PCa. In plasma, this panel demonstrated both a sensitivity and specificity of 72% (AUC = 0.72).

##### Diagnosis: Whole-Genome Methylomes from Independent Urine Cohorts for the Identification of New Diagnostic Biomarkers Useful for PCa

To overcome the challenge of validating biomarkers in liquid biopsies, methylation profiling (H450M BC) was directly performed on a small urine cohort (MeDIP and microarray analysis; grade ≥ 4 PCa patients *n* = 5 and controls *n* = 6), which identified 3986 DMCpGs (1464 DMGs) discriminating PCa patients from controls. After excluding unspecific targets that were also detected in patients with prostate removal, 196 candidates were retained, including *PLA2G16*, which showed strong discriminative potential. In tissues, *PLA2G16* expression (qRT-PCR) was inversely correlated with methylation (r = −0.46; *p* < 0.001). In both training (controls *n* = 43; tumor-associated samples *n* = 40) and validation (controls *n* = 50; tumor-associated samples *n* = 35) sets in the urine cohort, hypermethylation of six CpGs in the *PLA2G16* gene body was confirmed, yielding AUCs between 0.62 to 0.8. This translated to a sensitivity of 32% but a high specificity of 94%, while PSA alone reached only an AUC of 0.52 [46].

## 3. Prognosis

The identification a pre-metastatic PCa area is critical for avoiding metastasis and reducing the mortality rate. Conventional tools with biochemical and histological criteria (lymph node status, PSA level, and Gleason score) are still routinely used (for conventional techniques used in clinics, see the review [47]). The Gleason score, which requires an invasive examination, remains the most reliable criterion [48]. However, gene expression dysregulation that is frequently observed in DMGs may contribute to PCa carcinogenesis and represents a potential source of therapeutic targets. In addition, these DMGs may assist in patient stratification, grading, and recurrence prediction. Indeed, numerous laboratories have investigated whether cancer-related genes are subject to methylation-associated modifications. For example, MSP and WB analyses of DACT-2, a protein commonly downregulated in multiple cancers, confirmed in a small paired cohort (PCa/adjacent tissues *n* = 7) and PCa cell lines that its expression is directly controlled by DNA methylation [49]. Notably, DACT-2 OE (overexpression) in PC-3 cells reduced both cell migration and invasion, indicating that *DACT-2* silencing by methylation contributes to PCa progression. Based on its reported role in other cancers, NPTX2 expression was showed to be reduced in PCa compared with normal prostate tissues (TCGA RNAseq and methylome data; *p* < 0.05) in a methylation-dependent manner (r = −0.35; *p* < 0.001) [50]. Treatment with 5-azadC restored NPTX2 expression (mRNA and protein) in PC-3 and DU145 cells, while NPTX2 OE suppressed proliferation and impaired tumor growth in nude mice. Similarly, the loss of the expression of *AJAP1*, a putative TSG, has been associated with aggressiveness in several cancer types. TCGA methylome and transcriptome data demonstrated an inverse relationship between *AJAP1* expression and methylation in PCa [51]. Treatment with 5-azadC partially restored AJAP1 expression, which negatively impacted the EMT (epithelial–mesenchymal transition) pathway and tumorigenic phenotypes in PCa cell lines. Moreover, qRT-PCR and qMSP of paired tissues (PCa vs. normal adjacent tissues, *n* = 30) revealed decreased *miR195* expression associated with promoter hypermethylation. Treatment of PC-3 and DU45 cells (low basal *miR195* expression) with 5-azadC increased *miR195* levels and reduced proliferation, migration, and invasion, effects that were reversed by miR195 inhibition. *SLC15A2* gene body methylation was inversely correlated with its expression in PCa tissues (TCGA data) [52]. Knockdown (KD) of *SLC15A2* (siRNA) in 22Rv1 cells promoted both cell proliferation and migration, suggesting a TSG role. Similarly, *SLC14A1* repression was mediated by DNA methylation in PCa and correlated with poor PFS [53]. A combined TCGA analysis (PCa *n* = 126; controls *n* = 73) identified *FAM107A* as a methylation-silenced gene in PCa [54]. Low FAM107A expression was confirmed at the protein level in local cohorts (WB and IHC). In DU145 and PC-3 cells, FAM107A OE inhibited cell proliferation, migration, invasion, and tumor formation, while promoting apoptosis. *SLC16A5* also showed a strong inverse correlation between promoter methylation and expression (r = −0.8; *p* < 0.001; UALCAN data) [55]. Low expression correlated with the Gleason score and grade but not OS (AUC = 0.9). In addition, low *PGM5* expression and promoter methylation were linked to the Gleason score, a poor prognosis, or biochemical recurrence (TCGA). OE of PGM5 reduced the proliferation and migration of PC-3, LNCaP and DU145 cells [56].

EPHA5 was significantly downregulated at both the mRNA (qRT-PCR) and protein (WB) levels in PCa tissues [57]. In a Chinese cohort (PCa *n* = 22; PCa/adjacent paired tissues *n* = 23; BPH *n* = 39), *EPHA5* promoter methylation was inversely correlated with expression and associated with the grade and stage. Treatment with 5-azadC restored EPHA5 expression in PCa cell lines (LNCaP, PC-3, and DU145), while OE suppressed migration and invasion. Combined qMSP and qRT-PCR experiments on a Chinese cohort also revealed that *PCDH8* promoter methylation is specifically downregulated in PCa compared to BPH [58]. *PCDH8* methylation was significantly associated with the tumor size, stage, and grade (*p* < 0.05). In a Thai FFPE cohort (MSP; PCa *n* = 62; BPH *n* = 81), *SOX11* promoter methylation was more frequent in tumors than in benign samples (*p* < 0.001) [59] and was associated with the Gleason score, peri-neural invasion, and PSA level (*p* < 0.05) [60]. Similarly in another Thai study (MSP; PCa *n* = 92; BPH *n* = 62), *PAQR3* promoter methylation occurred more frequently in tumors than in benign tissues (*p* < 0.01) and was correlated with peri-neural invasion (*p* < 0.05) [60]. *SPARC* methylation (MSP) was observed in 145/207 PCa samples but only 1/38 prostate controls (absent in PBMCs *n* = 30). It was associated with lymph node metastasis, advanced stages, and a higher Gleason score (*p* < 0.01), suggesting its value as a prognostic marker [61].

*CRMP4* promoter methylation was significantly associated with the tumor grade (OR = 1.95; *p* < 0.001) in both uni- and multivariate analyses. In FFPE samples (pyrosequencing *n* = 631) [62], a cutoff of 18% methylation discriminated high-risk tumors (Gleason score ≥ 8) with excellent performance (sensitivity = 87%; specificity = 99%; AUC = 0.93), outperforming biochemical markers alone. Concordant results were obtained with frozen samples from a large Chinese cohort (pyrosequencing; high-risk PCa *n* = 230; intermediate-risk PCa *n* = 30), where *CRMP4* promoter methylation independently predicted PFS (*p* < 0.001). Huang et al. (2018) demonstrated that combining CAPRAS with the *CRMP4* methylation status (cutoff 15%) predicted PFS with 60% and 80% accuracy at 3 and 5 years, respectively [63]. Large validation cohorts confirmed this: pyrosequencing of samples from Chinese (PCa *n* = 339) and German (PCa *n* = 328) cohorts identified *CRMP4* methylation as an independent predictor of lymph node metastasis (cohort 1 HR = 8.4; cohort 2 HR = 12.5; both *p* < 0.001) [64]. Importantly, targeted demethylation of the *CRMP4* promoter (TALEs in PC-3 cells) abolished metastasis in mouse models, confirming a functional role in aggressiveness [65].

*CXCL12* promoter methylation significantly distinguished cancer from benign and normal prostate tissues in an FFPE cohort (PCa *n* = 25; adjacent tissues *n* = 22, BPH *n* = 19; *p* < 0.05) [66]. In a large cohort (qMSP; PCa *n* = 247), *CXCL12* methylation correlated with the Gleason score (*p* < 0.001), nodal involvement (*p* < 0.05), and PFS (HR = 1.9–2.1; *p* < 0.05). The results were validated in TCGA database (PCa *n* = 498; controls *n* = 50), where *CXCL12* methylation remained significant in multivariate analysis. A multicenter study (Canada, Austria, Portugal, and Germany) also reported *TERT* promoter hypermethylation in PCa compared with control tissues (MeDIP-seq; PCa *n* = 51; control tissues *n* = 53; *p* < 0.001), which correlated with the Gleason score and grade (*p* < 0.01; *p* < 0.05). In a validation cohort (PCa *n* = 139), *TERT* methylation predicted PFS (*p* < 0.05) and was an independent prognostic factor for patients with a Gleason score of 6–7 (HR = 3.6; *p* < 0.05) [67]. *GFI1* promoter methylation occurred in 37–51% of PCa samples (bisulfite sequencing or MSP; *n* = 39), and was associated with poor PFS (*p* < 0.001) [68]. Treatment with 5-azadC restored *GFI1* expression in DU145, LNCaP and PC-3 cells, and reduced viability and tumor growth in nude mice, confirming its tumor suppressor function. A multicenter study showed that *SLC18A2* methylation was detected in TCGA database (H450M BC; PCa patients *n* = 297; controls *n* = 34) and a Danish cohort (PCa patients *n* = 19, and controls *n* = 11), where it inversely correlated with expression (*p* < 0.05) [69]. A validation cohort (PCa patients *n* = 280; CRPC patients *n* = 29; BPH patients *n* = 15; MPC patients *n* = 31; PIN patients *n* = 11; controls *n* = 18) confirmed its association with the methylation Gleason score, T-stage, and PSA recurrence (HR = 1.8–2.3; *p* < 0.05, univariate analysis). Similarly, *CDO1* expression (qRT-PCR) was regulated by promoter methylation (qMSP) in paired tissues from German patients (PCa/adjacent paired tissues *n* = 16; *p* = 0.003) and in TCGA (r = −0.6; *p* < 0.001) [70]. Hypermethylation correlated with the Gleason score, Ki-67 proliferation index, and biochemical recurrence (HR = 1; *p* = 0.002), in a large German validation cohort (qMSP *n* = 300); although performance was lost in multivariate analysis (HR = 2.1; *p* < 0.05). *OLFM4* expression was negatively correlated with PFS [71]. In pyrosequencing analyses (low-grade PCa *n* = 6; middle- to high-grade PCa *n* = 8; normal adjacent tissues *n* = 8), 3/8 CpGs were hypermethylated in high-grade tumors, and inversely correlated with protein expression (IHC). 5-azadC restored OLFM4 expression in PC-3 and RWPE1 cells, while RNAi knockdown promoted the expression of EMT markers and reduced tumor growth in mice, confirming functional involvement. *SRD5A2* promoter methylation predicted OS (*p* = 0.001) and PFS (*p* = 0.003) (bisulfite sequencing; PCa *n* = 42; mCRPCa *n* = 12; BPH *n* = 36), with a cut-off of 37,9%. Multivariate analysis confirmed the independent association with OS (*p* = 0.03) for this biomarker [72]. *PD-L1* methylation analysis in TCGA (PCa *n* = 498, adjacent tissues *n* = 65) revealed that a single specific CpG site was inversely correlated with expression and predicted recurrence (HR = 2.6; *p* = 0.001) [73]. A German cohort (qMSP; PCa *n* = 299) validated these findings for biochemical recurrence and PFS (*p* < 0.05). Finally, *BRCA1*, a target that has been well established in breast and ovarian cancers, was also silenced by promoter hypermethylation in PCa (23/30 PCa tissues, 0/10 control tissues). *BCAR1* promoter methylation correlated with an advanced stage (*p* = 0.01) and higher Gleason score (*p* = 0.007) [74].

*PITX2* methylation discriminated cancer from control tissues (*p* < 0.001), predicted recurrence (HR = 1.8; *p* < 0.05), and was associated with PFS in a German cohort (qMSP; PCa/adjacent paired tissues *n* = 24; PCa *n* = 300; PCa biopsies *n* = 32; BPH *n* = 31) [75]. A meta-analysis of seven studies (PCa *n* = 2185) confirmed its prognostic value for relapse (HR of 2.7; 5-year BCR-free survival: high vs. low *PITX2* methylation: 71% vs. 90%) [76]. In a Chinese cohort (qMSP PCa *n* = 44 and BHP *n* = 43), *PITX2* methylation correlated with higher stages (*p* < 0.05) [77]. Concerning *PITX3* promoter methylation (TCGA; PCa *n* = 498, controls *n* = 50), it identified tumors vs. control tissues (*p* < 0.001) and correlated with the Gleason score, stage, and baseline PSA level (*p* < 0.05). It predicted biochemical recurrence (HR = 1.83; *p* < 0.05) and PFS [78]. FFPE samples from a large German cohort (qMSP *n* = 300) confirmed recurrence and PFS predictions (HR = 2.5; *p* = 0.001).

Conversely, some genes are upregulated through promoter hypomethylation, which may drive tumor aggressiveness and hold prognostic value in PCa. *STEAP1*, which is suspected to act as oncogene, promotes cell proliferation and invasion, and its expression is elevated in several cancers, including PCa. Analysis of TCGA data (RNAseq and M450K BC) confirmed that increased *STEAP1* expression is associated with promoter hypomethylation in PCa compared to normal prostate [79]. Similarly, EIF4A1 expression, which is frequently upregulated across cancers (15/24, including PCa), is negatively correlated with methylation (TCGA: r = −0.53; *p* < 0.001). High *EIF4A1* expression was also linked to reduced OS in PCa patients (*n* = 499; HR = 5.3; *p* < 0.0001) [80]. In addition, *Line-1*, a repetitive element reflecting global DNA methylation, is commonly hypomethylated across cancers [81]. In PCa, its hypomethylation was confirmed in an Italian cohort (PCa/adjacent paired tissues *n* = 152) compared to controls and was significantly associated with OS (HR = 1.4).

### 3.1. Prognosis: Whole-Genome Methylomes from Public Databases for the Identification of New Prognostic PCa Biomarkers

Using methylomes from public databases, several unsupervised analyses have aimed to identify novel candidate DMCpGs predictive of recurrence or survival of PCa patients. Methylation profiles from TCGA (PCa *n* = 73; benign *n* = 63) initially revealed 564 DMGs, including 87 DMGs previously linked to recurrence [82]. *ZNF154* emerged as the top candidate, with Kaplan–Meier analyses confirming that methylation of three specific CpGs was significantly associated with PFS (*p* < 0.05) in both univariate and multivariate models. In TCGA data (H450K BC; PCa and RNAseq *n* = 530 and normal tissues = 50), 11,255 DMCpGs were detected in *lncRNA* promoters (within −2000 from TSS), among which 484 CpGs showed an inverse association with expression. Of these, 48 DMCpGs were associated with OS. A LASSO-derived methylation score based on sixteen CpGs (eight hypomethylated and eight hypermethylated CpGs), predicted OS at different time points with high accuracy (AUC: 0.89, 0.92 and 0.93 at 3, 5, 7 years; *p* = 0.001). Similarly, an analysis of the GEO methylome (PCa *n* = 31; normal prostate *n* =16) by Tonmoy et al. (2022) [83] identified 2622 DMCpG, including 566 sites in *lncRNA* promoters, supporting a substantial role of epigenetic regulation in PCa carcinogenesis. Integration of methylation and expression data revealed eight DMCpGs with inverse expression–methylation relationships: four upregulated via promoter hypomethylation and four downregulated via promoter hypermethylation. All four methylation changes were significantly associated with a poor prognosis and OS of PCa patients. Further GEO analysis (PCa tissues *n* = 73; adjacent prostate tissues *n* = 63) identified 10,206 DMCpGs in 2182 promoters, including 4 specific CpGs in the *SLCO4C1* promoter that were strongly negatively correlated with gene expression (r = −0.2 to −0.4, *p* < 0.001) [84]. *SLCO4C1* promoter methylation was validated in TCGA (PCa/adjacent *n* = 50). Three of the four DMCpGs were significantly associated with the Gleason score or biochemical recurrence. Multivariate analysis confirmed these CpGs as independent predictors of PFS, establishing *SLCO4C1* methylation as a robust prognostic marker. In a Gleason score-based analysis, Geybels et al. (2016) identified 52 DMCpGs in TCGA (*n* = 333) discriminating low-grade (<6) from high-grade (8–10) PCa [85]. A virtual validation in TCGA (H450K BC; PCa *n* = 565) showed that each 25% increase in the methylation panel score was significantly associated with biochemical recurrence (HR = 1.78). The panel remained significant in multivariate models (including the Gleason score, stage, and PSA level), and the combined model achieved the best predictive performance for PFS (AUC = 0.78; *p* < 0.001). Using a machine learning approach on TCGA database (DNA methylation and RNA-seq), Aldakheel et al. (2025) identified 684 DMGs and 691 DEGs between recurrence and non-recurrence groups, including 10 genes (*AOX1*, *CCND1*, *COL5A3*, *FAMM71F2*, *FGFR1*, *SPIN2*, *SLC17A2*, *TNNI2*, *RNF169*, and *RREB1*) with inverse methylation–expression relationships, implicating them in PCa relapse [86]. Among these, *AOX1* downregulation by DNA methylation was validated at both the mRNA (TCGA RNAseq, H450KBC) and protein levels in PCa tissues and cell lines [87]. Moreover, 5-azadC treatment restored AOX1 expression and reduced PCa cell migration and invasion. Another TCGA study (PCa *n* = 480) also identified 241 DMCpGs that distinguished recurrent from non-recurrent cases [88]. A methylation score based on 11 CpGs achieved high predictive accuracy for biochemical recurrence (AUCs: 0.83, 0.72, and 0.71 at 1, 3, and 5 years, respectively).

At the transcriptomic level, TCGA (PCa *n* = 499; controls *n* = 52) revealed 567 DEGs [89]. EPCAM, which is markedly upregulated at both mRNA and protein level (IHC) in PCa, was negatively associated with the T-stage, Gleason score, and OS (AUC = 0.62 to 0.76 at 1 to 5 years). Notably, methylation of five specific CpGs associated with *EPCAM* showed a negative correlation with expression and four of them were significantly linked to PFS.

### 3.2. Prognosis: Independent Methylomes Reveal Prognostic Signatures in PCa

Several independent cohort-based studies have provided valuable insights into DNA methylation changes associated with prostate cancer, beyond the large public dataset such as TCGA. In a cohort of AA men (M850K BC, PCa patients *n* = 63; controls *n* = 50) 1655 CpGs were found to be associated with the Gleason score, among which 25 were inversely correlated with gene expression [90], suggesting their potential roles in tumor progression. Similarly, Liu et al. (2022), by comparing microarrays and H450K methylation data in a small cohort of 10 patients (localized PCa *n* = 4 and locally advanced PCa *n* = 6), identified 30 DMGs inversely correlated with expression and enriched in DNA replication and cell regulation pathways, supporting their relevance as biomarkers of PCa progression [91]. Further large-scale analyses have refined these observations. In a Swedish cohort (H450K BC; 12 PCa/adjacent tissues *n* = 12, bone metastasis *n* = 14, samples from short-term castrated patients *n* = 4, and mCRPCa samples *n* = 52) 4 360 CpGs discriminated PCa from adjacent normal tissues, 14 875 distinguished metastases from PCa, and 1183 DMCpGs separated metastatic subgroups. Importantly, hypermethylation predominated (86% in PCa tissues vs. normal tissues, 88% in metastases vs. PCA), including androgen receptor (AR) hypomethylation that is specifically linked to bone metastasis [92].

In parallel, an Australian cohort enriched in high Gleason score tumors (M450K BC, PCa tissues *n* = 125, adjacent tissues *n* = 41, control tissues *n* = 2) revealed 2386 DMCpG discriminating benign tissues from PCa [93]. Data normalization agreed with a previous study at 95% in a North American cohort [18]. A LASSO-based panel of 16 CpGs (*cg11213697*, *cg21918559: intergenic)*, *ARL9*, *B3GNLT1*, *CLEC14A*, *CYP2W1*, *DTX4*, *EGFL6*, *FAM115A*, *GSTM2*, *LOC100289511*, *MC5R*, *PRKCB*, *SFRS5*, *STK31*, *SYNGR1*, and *USP44*) achieved a striking predictive accuracy (AUC = 0.99). Interestingly, *GSTM2*, *PRKCB*, and *USP44* methylation and expression were negatively associated in this panel. A second panel could also stratify patients based on the Gleason score (*cg05364411*, *cg19419246*, *cg27192635*, and *cg22287731*: intergenic, *FCGBP*, *FREQ*, *MAGI2*, *TRIP13*, and *WWOX*) but this could not be confirmed in other cohorts (TCGA and FHCC databases). However, validation across external dataset yielded inconsistent results, underscoring the difficulty of transferring biomarker panels between independent cohorts. Other studies have emphasized prognostic relevance. In a Danish cohort (H450K BD/RNA-seq; PCa patients *n* = 20, controls *n* = 13), 1125 DMGs were shown to be regulated by methylation, among which *MEIS2* was significantly associated with the Gleason score, tumor stage, and PFS [94]. While *MEIS2* methylation was correlated in TCGA (r = −0.67; *p* < 0.001), it did not retain independent prognostic value in multivariate models. At the clonal level, methylome analyses of paired primary PCa and lymph node metastases (H450K BC; PCa/lymph node metastasis paired tissues *n* = 16) highlighted intratumoral heterogeneity, revealing polyclonal origins in 50% of cases and showing that metastatic dissemination was often linked to a dominant subclone [95].

The prognostic utility of methylation panels has also been demonstrated in larger cohorts [96]. (H450K BC; PCa patients *n* = 215 and controls *n* = 404) showed that hypermethylation of genes such as *ADM*, *AEN*, *CCND1*, *CDC2*, *ORC2L RAP1GAP*, *RASSF1A*, and *TGFB1* was associated with a reduced risk of metastasis, whereas hypomethylation of *CPN1*, *RARB*, and *VCAM1* conferred higher risks [96]. The combination of methylation data with clinical scores slightly improved the predictive performance (AUC from 0.68 to 0.73; validated at 0.8). Among the DMGs associated with the metastasis prediction were *FAM66A*, *PRDM16*, and *TRPS1*, which have already been related to PCa carcinogenesis [97].

Likewise, another US study (training cohort PCa *n* = 344; mPCa *n* = 48) identified forty-two metastasis-associated CpGs (AUC 0.54 to 0.84) [98], eight of which were validated, and *ALKBH5*, *FHAD1*, *KLHL8*, and *PI15* improved the Gleason-based risk stratification. Smaller independent analyses also support the role of *WNT5A* hypomethylation and overexpression in metastatic progression [99].

At the recurrence level, a Spanish cohort (GoldenGate methylation Cancer Panel; PCa patients *n* = 58; controls *n* = 10) showed 1505 hypermethylated CpGs (located in 807 different promoters), with a methylation score based on the top 28 DMGs predictive of biochemical recurrence [100]. Six genes (*ALOX12*, *APC*, *GSTM2*, *MT1A*, *MYCL2*, and *RARB*) were associated with recurrence, and two retained independent significance (HR = 2.7–3.8). Similarly, in AA patients (H450K BC; PCa *n* = 76), 11,444 DMCpGs correlated with recurrence and 23 robust biomarkers were identified, including *FLNA*, *SLC25A20*, and *TNXB* [101].

Finally, familial studies have highlighted potential epigenetic inheritance. Analysis of blood methylomes from 133 family members identified 41 heritable CpGs linked to the PCa risk [102]. Among them, nine CpGs located near VTRNA2-1 were strongly predictive of aggressive PCa, suggesting that intergenerationally stable methylation marks may contribute to familial predisposition.

### 3.3. Prognosis: Whole-Genome Methylomes from Public Databases for the Identification of New Prognostic PCa Biomarkers and Independent Validation

Methylomes obtained from the Marmal-Aid database ((H450K BC; PCa *n* = 187; normal prostate *n* = 81; other cancers *n* = 2294; other normal tissues *n* = 634; blood cells *n* = 876) enabled the identification of seven DMCpGs specifically associated with PCa and absent in PBMCs, while four additional DMCpGs appeared as potential pan-cancer biomarkers [103]. These markers were subsequently validated in a small Danish cohort (qMSP; PCa *n* = 16; adjacent tissues *n* = 19; PBMs *n* = 40), where eight of the eleven candidates displayed sensitivities above 75% and specificities of at least 84%, without any false-positive PBMCs. A larger validation cohort (qMSP; PCa patients *n* = 197, BPH patients *n* = 9, controls *n* = 28) further confirmed the high diagnostic performance of this panel (AUC > 0.88 for each marker). Importantly, four of them (*DOCK2*, *GRASP*, *HIF3A*, and *PFKP*) significantly predicted biochemical recurrence and PFS in both univariate and multivariate analyses (HR: 1.96–18.78, *p* < 0.05).

To specially identify markers of aggressiveness, methylomes of intermediate- to high-Grade PCa were analyzed in an American cohort (CHARM arrays; PCa GS8 *n* = 6, PCa GS > 7 *n* = 8, adjacent tissues *n* = 10). This study revealed 913 DMGs, among which the top 6 (*ELAVL2*, *EXT1*, *IRX5*, *FLRT2*, *MAB21L1* and *OPCML*) efficiently discriminated between low- and high-Grade patients [104]. Validation using pyrosequencing confirmed *OPCML* and *FLRT2* methylation as robust markers in an independent cohort (high Gleason score *n* = 33, low Gleason score *n* = 20). Similarly, the methylomes of Danish patients (H450K BC; PCa *n* = 21; adjacent tissues *n* = 12; control tissues *n* = 9) identified 324 CpGs (163 genes) distinguishing PCa from control tissues [105]. After excluding well-established markers (e.g., *GSTP1* and *RARB*), a new panel of eight new DMGs was selected and validated (bisulfite sequencing; PCa patients *n* = 203, controls *n* = 30). All candidates efficiently predicted cancer (AUC: 0.79–0.91), while *COL4A6*, *PROM1*, *RHCG*, and *TCAF1* methylation were additionally associated with recurrence (*p* < 0.01). Notably, *RHCG* (HR = 1.6; *p* = 0.001) and *TCAF1* (HR = 1.5 *p* = 0.001) remained independent predictors in a multivariate analysis that included baseline PSA levels.

Whole-genome methylomes from TCGA (PCa *n* = 475) identified 1306 DMCpGs that stratified sample into four prognostic groups [106]. Groups 3 and 4 were associated with a higher Gleason score, higher baseline PSA content, and advanced-stage disease. Independent analyses of two large external cohorts (Fred Hutch *n* = 458; Canada ICGC *n* = 236) confirmed the prognostic risk score and highlighted the strong association of group 4 with metastatic potential. Complementary results from the GEO methylome (PCa/adjacent paired tissues *n* = 9) analysis also showed 6 899 DMGs (FC > 1.2), among which 411 robustly predicted biochemical recurrence [107]. Testing 10 of these DMGs in tissues (qMSP; PCa *n* = 151, BPH *n* = 17 and control tissues *n* = 51) confirmed the significant hypermethylation of *ADMATS12*, *NAALAD2*, and *PRKCB* in PCa compared to BPH (*p* < 0.05). Moreover, all three markers were associated with biochemical recurrence (*p* < 0.01). Importantly, combining the *PRKCB* promoter methylation status with PSA levels yielded a highly accurate predictor of recurrence (*p* < 0.001), whereas the PSA level alone was not informative.

### 3.4. Prognosis: Whole-Genome Methylomes from Independent Cohorts for the Identification of New Prognostic PCa Biomarkers and Independent Validation

RNAseq of paired tissues from a small Chinese cohort (PCa/adjacent tissues *n* = 10) identified 21 downregulated genes previously implicated in PCa carcinogenesis [108]. Among these, *TWIST2* expression showed a marked decrease in expression, which was further validated in TCGA transcriptomes (PCa *n* = 494, control tissues *n* = 106) and at the protein level in a local cohort (IHC, PCa/adjacent tissues *n* = 67). A significant inverse correlation was observed between *TWIST2* expression and methylation at specific CpGs. A functional study demonstrated that *TWIST2* OE in LNCap cells reduced cell proliferation and tumor growth in mouse xenograft models. Collectively, these finding support a tumor suppressor role for both *FAM107A* and *TWIST2* in PCa, with their methylation statuses serving as potential prognostic biomarkers.

Methylome profiling of an American cohort (H850K BC; aggressive PCa and paired control tissues *n* = 11, indolent PCa and paired control tissues *n* = 13) revealed 945 DMCpGs separating PCa from adjacent tissues [109]. A stringent selection (B-value cut-off of 0.2, *p* < 0.001) retained 17 DMCpGs, including *FAM71F2*, which was previously proposed as metastasis risk marker in testicular cancers. This suggests that diagnosis-related DMGs may also hold prognostic significance. Deep bisulfite sequencing of localized PCa stratified by clinical outcome (alive at 10 years post-RP, *n* = 4; deceased within 10 years post-RP, *n* = 4) identified 1 420 DMRs, of which 92% were hypermethylated in the deceased group [110]. After prioritization of the most discriminant DMRs, exclusion of signals detected in normal prostate and blood, and validation in TCGA, a panel of 18 DMRs was established. Testing in a validation cohort of localized PCa (*n* = 185) revealed that 5/18 DMGs (*AC074091*, *CACNA2D4*, *MARCH6*, *PRDM8*, and *ZNF655*) were associated with biochemical recurrence (BCR) and patient survival. Moreover, a multivariable analysis identified the best predictor of BCR as combined assessment of *CACNA2D4* methylation, Gleason grade, and baseline PSA level.

### 3.5. Prognosis: Validation and Prognostic Value of DMG Methylation Panels in PCa

Building on previous evidence that *ST6GALNAC3* and *ZNF660* methylation may be putative biomarkers of PCa [111], their clinical performance was evaluated in a large and heterogeneous cohort (qMSP; PCa *n* = 169, precancerous lesions *n* = 10, BPH *n* = 13, mPCa *n* = 15, CRPC *n* = 7 and adjacent prostate tissues *n* = 20) [112]. At 100% specificity, *ST6GALNAC3* and *ZNF660* methylation showed sensitivities of 70 and 69%, respectively, in distinguishing PCa from benign samples or control tissues. Moreover, *ZNF660* methylation was significantly associated with recurrence and PFS (*p* < 0.01), highlighting its potential prognostic utility.

Beyond individual genes, multi-marker methylation panels have also been developed to improve risk stratification. An eight DMG panel tested in two American cohorts (pyrosequencing; PCa *n* = 11 and mPCa *n* = 23) [98] identified *PI15* hypermethylation together with hypomethylation of *ALKBH5*, *ATP11A*, *FHAD1*, and *KLH8* as predictors of metastatic disease. This panel allowed the establishment of a risk score that outperformed the Gleason score in predicting outcomes (AUC = 0.91 vs. 0.87). Similarly, a three-DMG panel (*APC*, *GSTP1*, *RASSF1A*) assessed in two large fused cohorts (qMSP; *n* = 803) demonstrated its ability to identify high-Grade tumors (Gleason core > 7) with moderate accuracy (AUC = 0.66, *p* = 0.001) [113]. A four DMG panel (*APC*, *CRIP3*, *HOXD3*, and *TGFB2*), validated in a cohort of 101 PCa patients, showed higher methylation in larger FFPE biopsies than in small samples, suggesting that a minimum DNA threshold is required for robust testing [114]. When combined with the PSA level, this methylation panel improved the recurrence prediction (sensitivity of 79%, specificity of 65%, AUC = 0.71).

Among individual candidates, *GSTP1* methylation (qMSP) has consistently emerged as a powerful biomarker across multiple cohorts. In a German cohort (12/20 PCa), *GSTP1* methylation reached a sensitivity of 60% and specificity of 91% [115], and identified cancer cells even in histologically negative surgical margins, suggesting its potential for early detection. Its methylation status correlated with the stage, grade, lymph node involvement, and biochemical recurrence. A larger FFPE study conducted in Germany and Belgium (high-risk PCa *n* = 218; BPH *n* = 42) confirmed that although a five DMG panel (qMSP; *APC*, *CCND2*, *GSTP1*, *PTGS2*, and *RARB*) displayed higher promoter methylation in high-risk PCa, only *GSTP1* methylation remained significant in predicting metastasis or recurrence in multivariate analyses (HR = 3.7–4.3) [116].

Long-term patient follow-up studies (bisulfite sequencing, *n* = 15; median of 19.5 y) further confirmed that *GSTP1* methylation arises early in low-Grade lesions (PCa *n* = 23, precancerous lesions *n* = 18, proliferative inflammatory atrophy *n* = 37) and expands across CpG islands with disease progression, correlating with recurrence and mortality.

Additional multi-gene panels have also been proposed for the prognosis. A five DMG panel (pyrosequencing; *APC*, *GSTP1*, *RASSF1*, *RUNX3*, and *TNFRF10c*) discriminated low- (*n* = 22), intermediate- (*n* = 22), high-risk (*n* = 27) patients with high accuracy, although only *APC* methylation was independently predictive of biochemical recurrence (*p* = 0.005) [117]. A four DMG panel (qMSP; *APC*, *CRIP3*, *HOXD3*, and *TGFB2*), which was validated in two Canadian cohorts (*n* = 453), confirmed its predictive value for biochemical recurrence [118]. Its performance was time-dependent (AUC = 0.60 at 1.5 years vs. significant prediction at 5 years, *p* = 0.03). Integration of the methylation status with PSA or CAPRA scores significantly enhanced the prognostic performance. Likewise, a six DMG panel (*CCND2*, *DPYS*, *HSPB1*, *MAL*, *PITX2*, and *TIG1*) in a UK cohort (*n* = 352) provided superior OS prediction compared to the CAPRA score (HR = 2.7; *p* < 0.001) [119], with further gains when both clinical and epigenetic features were combined.

However, population-specific effects have also been reported. A five DMG panel (*APC*, *CCND2*, *MGMT*, *RARB*, and *RASSF1A*) tested in a large American cohort (*n* = 332) showed that only *APC* methylation significantly predicted recurrence, and only in white patients (*p* = 0.006) but not in AA men (*p* = 0.34) [120]. This highlights the importance of ethnicity in biomarker performance and the possible need for population-specific panels. Finally, a 10 DMGs panel (*AOX1*, *CCDC181* (C1orf114), *GABRE*, *GAS6*, *GSTP1*, *HAPLN3*, *KLF8*, *MOB3B*, and *SLC18A2*) efficiently discriminated tumors from controls in American and Danish biopsy cohorts, with individual AUCs ranging from 0.8 to 0.98. A simplified panel (*AOX1*, *GSTP1*, *HAPLN3*, and *SLC18A2*) also moderately predicted metastatic progression (qMSP; PCa *n* =67, mPCa *n* =66, controls *n* = 40) [121]. A methylation score obtained with a shorter panel was associated with the metastasis prediction in this cohort with high specificity (100%) but limited sensitivity (31–41%).

Taken together, these studies establish that DNA methylation of individual genes such as *GSTP1*, *APC*, or *ZNF660*, and, more effectively, multi-gene panels can provide clinically relevant information for PCa diagnosis, recurrence prediction, and risk stratification. Nonetheless, variability across sample types, ethnic groups, and technical thresholds underscores the necessity for large, standardized multicenter validations before their routine clinical implementation.

### 3.6. Prognosis: Performance Tests of PCa Biomarkers in Liquid Biopsies

#### 3.6.1. Prognosis: Value of Liquid Biopsy-Derived Methylation Biomarkers in PCa

As highlighted above, liquid biopsies represent a promising approach for the identification of non-invasive DMGs, not only for diagnostic purposes but also for prognostic assessments of PCa. Several serum- and plasma-derived biomarkers have already shown significant clinical potential. For example in serum samples from a Chinese cohort (MSP; early PCa *n* = 167; BPH *n* = 44) [122], *PCDH17* promoter methylation was detected in 57% of PCa patients and absent in BPH patients (*p* < 0.001). Its methylation was strongly associated with the stage, Gleason score, PSA level (*p* < 0.05), and biochemical recurrence (*p* < 0.001). Importantly, this epigenetic alteration was identified as an independent predictor of PFS in a multivariate analysis. Comparable results were obtained for *PCDH8* and *PCDH10*, whose methylation correlated with higher tumor stages, baseline PSA level, lymph node metastasis, and recurrence (*p* < 0.05), also acting as independent prognostic biomarkers in serum-based cohorts (*p* < 0.001–0.05) [123,124].

At the genomic level, *USP44* promoter methylation was inversely correlated with expression in TCGA data (H450K BC *n* = 340, controls *n* = 49, RNAseq) and linked to poor PFS (*p* = 0.001) [125]. Functionally, *USP44* silencing was associated with increased chromosomal instability and frequent inactivation of *PTEN*, underscoring its tumor-suppressive role in PCa carcinogenesis. Clinically, *USP44* methylation was absent in healthy donors and patients with localized PCa but detected in over half mPCa cases (qMSP; HD *n* = 0/10; early PCa *n* = 0/32; mPCa *n* = 20/39), where higher methylation levels were associated with reduced OS (*p* = 0.008) [126]. Similarly, *DACT-2* promoter methylation was more frequent in serum collected from PCa patients compared to those with benign disease or controls (qMSP; PCa patients *n* = 64; BPH patients *n* = 22; HDs *n* = 47) [49], showing strong correlations with the Gleason score and nodal involvement, and outperforming PSA levels in specificity (75% vs. 59%).

High-throughput methylome profiling further expanded the repertoire of candidate biomarkers. *SRD5A2* and *CYP11A1* methylation were identified by MBD sequencing experiments as a predictors of recurrence in PCa samples (PCa patients *n* = 86) [127]. A panel of seven DMCpGs (*ACSS3*, *CRABP2*, *DHRS4L2*, *NKX2-6*, *SCGB3A1*, *HOXA7*, and *SERPIN1B*) was progressively methylated from benign lesion to PCa (M450K BC; localized PCa *n* = 6 and adjacent *n* = 6) was later refined into smaller clinically adaptable panels [128]. When tested in a plasma cohort from patients with different grades (qMSRE; benign tumor patients *n* = 4; patients with localized PCa *n* = 65; mPCa patients *n* = 61; cut-off of *p* < 0.01 and 10-fold cross-validation) 87/92 of this large panel efficiently separated grades (AUCs = 0.95–0.97). A three-gene panel (qMSRE; *CHST11*, *CUGBP2*, and *PCDHGC4*) achieved near perfect discrimination of metastatic disease (AUC of 0.98), while a two-gene panel (*CRABP2* and *TNFAIP8*) predicted OS, albeit with reduced power in multivariate models including PSA levels (*p* = 0.04 and 0.02). Furthermore, a 15-gene signature, including *PROM1* and *CHST11* methylation, robustly predicted recurrence, with most markers retaining independent significance in multivariate analyses (HRs = 3.1–6.8) [128].

Liquid biopsy approaches have also been extended to exosome-derived DNA and circulating tumor cells (CTCs). In mCRPC, *GSTP1*, *RASSF1A*, and *SLFN11* promoter methylation (using qMSP) were consistently detected in both exosomes and EpCAM-positive CTCs (*n* = 61), but absent in healthy donors (*n* = 10) [129]. Concordant results were obtained for these two techniques of isolation, showing high methylation signal in samples issued from mCRPC patients (between 23 to 30% of methylation in CTCs vs. 29 to 40% in exosomes and no signal in HDs). *GSTP1* and *RASSF1A* promoter methylations were further correlated with poor OS (HR = 3.9, *p* = 0.017 and HR = 6.1, *p* < 0.01; respectively) in exosome samples. Advances in ultra-sensitive detection technologies such as OBBPA-ddPCR (optimized bias-based pre-amplification) highlighted the importance of pre-amplification to detect rare methylated fragments, improving sensitivity from 25% to 75% [130].

Finally, an optimized biomarker discovery pipeline developed by Trier Bjerre et al. (2020) screened TCGA and Marma-Aid methylomes (M450K BC; PCa *n* = 187, normal prostate *n* = 81, blood cells *n* = 876) to propose candidate markers useful for identifying clinical conditions and mPCa [131]. These DMGs were next evaluated in a small plasma cohort (qMSP; collected from PCa patients *n* = 16, BPH patients *n* = 19, and HDs *n* = 40) and due to unspecificity or technical limitations, 12 remaining DMGs were next tested for cancer specificity (qMSP-ddPCR; blood cells *n* = 44 and HDs *n* = 64). After several validation rounds, a final three-gene panel (MSP-ddPCR; *DOCK2*, *FBXO30* and *HAPLN3*) achieved high sensitivity and specificity in tissues (90–100%), but limited detection rates in a large plasma cohort (localized PCa *n* = 102, mPCa *n* = 65, BPH *n* = 61 and HDs *n* = 36). Notably, methylation positivity was enriched in plasma from patients with mPCa with high volume-associated tumors (71–89% vs. 19–32%), and correlated with disease progression (HR = 3.1; *p* = 0.001) and the Gleason score, suggesting a role for patient stratification for advanced disease.

Altogether, these studies underline the major potential of liquid biopsy-based methylation assays for non-invasive prognostic assessments of PCa. While several candidate biomarkers and panels demonstrated high discriminative power, especially in metastatic settings, their translation into the clinical routine still requires the standardization of detection methods, validation in large prospective cohorts, and integration with established clinical parameters such as PSA levels.

#### 3.6.2. Prognosis: Whole-Genome Methylomes from Plasma Samples for the Identification of New Prognostic PCa Biomarkers

To overcome the challenges of translating efficient tissue-derived prognostic biomarkers of prostate cancer (PCa) into liquid biopsy assays, several laboratories have attempted to directly identify differentially methylated genes (DMGs) in plasma or urine. A comparative analysis of methylomes from mPCa patients (450K BC; mPCa/adjacent paired tissues/paired urine/paired blood *n* = 4) demonstrated that methylation profiles were highly correlated between plasma and urine within individual patients (r = 0.93). However, liquid biopsy-derived profiles more closely resembled matched control tissues (r = 0.81) than tumor tissues (r = 0.5), suggesting that most DMGs detected in plasma or urine may not originate directly from tumors [132].

In another study, Wu et al. (2020) [133] analyzed plasma methylomes by deep bisulfite sequencing (baseline mCRPCa *n* = 19; progressing mCRPCa *n* = 16; HD *n* = 2; blood cells *n* = 15). They identified the top 1000 DMRs associated with clinical outcomes [133]. GSEA filters revealed that targets of Polycomb repressor complex 2 (PRC2) were strongly linked to the metastatic prediction in plasma (*p* < 10^−4^). Similarly, Chen et al. (2022) performed MeDIP-seq on plasma samples from localized PCa (*n* = 30) and mCRPCa patients (*n* = 130) [134]. They reported widespread hypermethylation of TSGs in mCRPCa patients compared to primary PCa patients, while promoter methylation of oncogenes remained largely unchanged. Using machine learning approaches, a panel of the top 150 hypermethylated and 150 hypomethylated TSGs achieved an AUC of 0.99 for distinguishing mCRPCa from primary PCa. Among these, *NR3C1* promoter hypermethylation (coding GR) emerged as the most robust discriminator. Notably, no *NR3C1* methylation was detected in leukocytes from HDs. Functional enrichment analysis suggested that *NR3C1* methylation was associated with the downregulation of genes involved in antigen presentation, pointing to impaired immune performance in aggressive PCa.

#### 3.6.3. Prognosis: Performance Tests of PCa Biomarker Sets in Urine

*GSTP1* and *APC* methylation statuses significantly increased performance for the identification of high-risk patients compared to the Gleason score alone (AUC = 0.89) in urine samples from a multicenter American cohort (qMSP; *n* = 96) [135]. Similarly, methylation of at least one out of three DMGs (*GSTP1*, *RARB*, and *RASSF1A*) was identified in 80% of urine samples collected from two (voided urine or catheterized samples) Lithuanian cohorts of PCa patients (qMSP; *n* = 514), but only *GSP1* methylation presented significant methylation differences according to the tumor stage and only in the voided cohort [136]. This result underlines the importance of the choice of the clinical protocol for diagnosis. Although the methylation score failed to predict the stage, the consideration of the PSA level clearly improved specificity (from 28% with PSA levels alone to 65% in the combined test) but dramatically decreased sensitivity (from 87% with PSA levels alone to 57%). A multicenter study (USA, Canada, Ireland, and England) aimed at testing the methylation performance of a six DMG panel (*APC*, *GSTP1*, *IGFBP3*, *IGFBP7*, *PTGS2*, and *SFRP2*) [137]. They first validated the hypermethylation status of these biomarkers in two independent methylome (H450K BC) tissue cohorts (own cohort PCa *n* = 21 BHP *n* = 10; TCGA PCa *n* = 144 BHP *n* = 34) and next in a large set of urine samples (qMSP *n* = 319) collected from PCa patients. Although the methylation score gave a similar AUC value to the PSA level (0.64) to detect PCa in urine, the combination of both criteria predicted high-grade PCa very efficiently (AUC = 0.95). In two complementary studies, Brikun et al. also tested 32 DMGs (19 in study 1 and 13 in study 2) in the same urine samples (semi-quantitative MSP; *n* = 94) [41,138]. Some targets, such as *GPR62*, *HOXD3*, *HOXA7*, or *KLK10*, could effectively detect early PCa in urine confirming these markers as good candidates for diagnosis. Other DMGs were also more frequently observed in urine from patients presenting higher Gleason scores, suggesting that these markers could be more helpful for determining the prognosis using urine.

Based on their roles in carcinogenesis and their frequent regulation by promoter methylation, 17 cancer-related DMGs were first tested for their diagnosis efficiency in urine samples collected from Ukrainian men (qMSP; PCa *n* = 31 and HD *n* = 33) [139]. The top 6 DMG panel (no methylation in controls, high methylation in cancer patients) was retained in a shorter panel (*APC2*, *CDH1*, *FOXP1*, *LRRC3B*, *WNT7A*, and *ZIC4*) whose methylation score in urine led to a sensitivity of 78% and specificity of 100% (score of 0–1 33/33 controls). Moreover, this methylation score showed a quite good correlation with the tumor stage (score of stage 1 patients: 0–1 vs. all scores of mPCa patients: ≥4).

### 3.7. Prognosis: Biomarkers for the NE Subtype

The diagnosis of CRPCa-NE (castration-resistant neuroendocrine prostate cancer) remains particularly challenging and still relies on metastatic biopsies. To identify specific DMCpGs for this aggressive phenotype, Berchuck et al. (2022) [140], first analyzed methylomes from cell models (MeDIP-seq; LuCaP PDX, NEPCa *n* = 5; PCa *n* = 24). They identified 76 NE-associated sites as well as 277 PCa-associated DMRs (*p* < 10^−6^ and log_2_FC > 3), none of which were present in leukocytes [140]. These NE-specific methylation signatures were subsequently validated in a plasma cohort (cfMeDIP-seq; NE-mCRPCa *n* = 11; non-NE-mCRPCa *n* = 45; *p* < 0.01), where a methylation-based score achieved 100% sensitivity and 90% specificity for the identification of NE-mCRPCa patients. Similar performances were confirmed in an independent validation cohort (NE-mCRPCa *n* = 16, non-NE-mCRPCa *n* = 57).

Consistent findings were also reported in earlier studies (CRPCa *n* = 18; CRCP-NE *n* = 10; normal prostate *n* = 7), which identified a distinct set of DMGs for each group [141]. Transcriptional factor motif enrichment analysis revealed that NE-CRPCa was associated with the hypermethylation of canonical PCa regulators (*AR*, *HOXB13*, and *REST*) and hypomethylation of neuroendocrine TF genes such as *ASCL1* and *NEURO1D*. Based on these observations, a 53-DMR panel, termed NEMO, was developed for the molecular identification of CRPC-NE. In cfDNA samples, stable phenotypic evidence (PE) was achieved when the tumor content exceeded 3%. Using this approach, the PE score successfully identified 79/82 CRPCa and 17/20 CRCP-NE patients, while no clinically confirmed non-NE patient was misclassified.

Finally, BCL2 expression, which is known to promote resistance to apoptosis in CRPC-NE, was found to be upregulated in these tumors. Interestingly, an analysis of PDX PCa cell lines revealed a correlation between *BCL2* expression and its promoter methylation, suggesting a methylation-dependent regulation that could represent a potential therapeutic vulnerability in this otherwise treatment-refractory disease [142].

### 3.8. Prognosis: Ethnicity and DNA Methylation in PCa

African American (AA) men present a higher risk of PCa than American men of European Ancestry (EAA). Genetic and epigenetic roles in PCa incidence in AA men is poorly understood since AA men are under-represented in cohorts. A recent review summarized studies comparing genetic and DMG differences between AA and EAA PCa patients [143]. Indeed, *PMEPA1*, *RARB*, *SNRPN*, and *TIMP3* appeared to be differentially methylated between AA and EAE men. A comparison of methylation profiles of PCa and adjacent tissues (76 AA men and 75 EAA men) revealed 76,400 DMCpGs that were common in both AA and EAA men (including 6267 in promoter regions) [144]. A panel of 10 DMCpGs was able to discriminate benign from PCa tissues (AUC > 0.9). Interestingly, 639 specific DMGs were specific to AA men and 1304 to EAA men, suggesting that ethnicity-related specific DNA methylation-mediated gene regulation may contribute to the higher incidence of PCa in AA men and that biomarker design should account for ethnicity. In a similar manner, a loss of expression of *ROBO1* has been reported to be much more frequent in AA PCa patients than in EAA PCa patients and is associated with a high risk of recurrence. This gene is frequently methylated in AA PCa patients, and the ROBO1 pathway could be an interesting strategy of treatment for this population [145]. Inactivation of the TSG *miR-34b* by DNA methylation was also more frequently observed in AA men than in Caucasian men with PCa, suggesting a putative role in PCa incidence in AA men [146].

An original approach analyzed the methylation of four DMGs in AA men with PCa (*n* = 65), which were characterized for an admixture of West African, Native American, and European ancestry [147]. Indeed, methylation of *NKX2-5* and *RARB2* correlated with African ancestry (r = 0.43; *p* = 0.001 and 0.29; *p* = 0.04, respectively) whereas it negatively correlated with European ancestry (r = −0.43; *p* = 0.001 and −0.28; *p* = 0.04, respectively).

## 4. Prediction of the Treatment Response

### 4.1. Prediction of the Treatment Response: Identification of New Markers

AR pathway inhibitors (ARis) are represent a cornerstone of advanced PCa treatment. However, their prolonged use frequently leads to the emergence of AR-independent CRPCa, often accompanied by a loss of AR expression. Increasing evidence suggests that DNA methylation alterations play a key role in therapeutic escape. Indeed, enzalutamide was shown to enhance DNMT activity and increase the global 5mC content in LNCaP and C4-2B cells [148], with similar findings observed in enzalutamide-resistant C4-2B-MDVR, supporting the concept that ARis may induce DNA methylation reprogramming and thereby contribute to treatment resistance. Comprehensive methylome analyses (450K BeadChip) of PCa cell lines recapitulating distinct disease stages (LNCaP, vehicle-treated LNCaP (V16D^CRPC^) and enzalutamide-resistant cell lines (with NE or PSA-secreting phenotypes) identified 507 DMCpGs associated with both CRPCa and ARi resistance [149]. Cross comparison with independent dataset confirmed 81 promoter hypermethylation events maintained in NE states, including *AR* promoter hypermethylation, which was specially linked to ARi exposure but not NE differentiation. In line with these observations, a Spanish cohort (methylome cancer panel; PCa patients with androgen deprivation *n* = 45 and controls *n* = 10) identified 61 DMGs associated with resistance to androgen deprivation therapy (ADT) [102], with *ETV1* and *ZNF215* hypermethylation efficiently predicting disease progression (HRs = 3.8 and 2.9, respectively). Furthermore, serial plasma analyses (PCa patients at baseline *n* = 55; 3 months after starting an androgen-deprivation therapy *n* = 55; before 24 months in the case of progression *n* = 15; or at 24 months for patients without progression *n* = 14) [150] revealed that baseline 5hmC (5 hydroxymethylcytosine) profiles could discriminate responders from low-responders, with a three-gene DhMG score (*AR*, *FOXA1*, and *GRHL2*) predicting progression with high accuracy, underscoring the clinical potential of 5hmC-based liquid biomarkers in monitoring the ADT response.

Resistance mechanisms also extend to radiotherapy. Activation of the IRAK1 pathway, linked to immune-dependent cell death, was associated with *IRAK1* CpG hypomethylation in PCa, correlating with increased expression (TCGA methylomes: PCa *n* = 341, controls *n* = 35; and RNA-seq) and H3K27ac enrichment [151]. In In-3 and LNCAP cells, pharmacological reactivation with demethylating agents (5-azadC) and HDAC inhibition further enhanced (fourfold) *IRAK1* expression, suggesting that its methylation status could guide the use of IRAK1-targeted adjuvant therapy (e.g., Jh-X-119-01 and pacritinib). In parallel, radioresistant PCa cell models (DU145 cells) revealed that *miR200c-3p* was silenced via hypermethylation [152], while restoring its expression resensitized cells to irradiation. Conversely, PTBP1 (polypyrimidine tract binding protein) OE, which is linked to poor outcomes [153], was shown to interact with RALY and modulate DNMT3B splicing, stabilizing the DNMT3B-L isoform and repressing *DUSP2* expression, ultimately driving radioresistance.

Additional mechanisms of resistance may also involve metabolic regulators. Thymidine kinase (TK1), which is consistently upregulated in PCa and associated with a poor prognosis [154], was shown to be epigenetically regulated. Integrative analyses of TCGA methylome and single cell transcriptome datasets indicated that *TK1* methylation not only governs its expression but also correlates with immune cell infiltration, suggesting that the *TK1* methylation status could serve as a predictive biomarker of immunotherapy responses, while simultaneously representing a potential therapeutic target.

### 4.2. Prediction of the Treatment Response: Performance of New Markers in Liquid Biopsies

*SHOX2* and *SEPT9* promoter methylation have been successfully developed as clinical cfDNA assays for lung and colorectal cancer (Epi proColon and Epi proLung). Given their potential as pan-cancer biomarkers, their utility in PCa has been explored (PCa positive for PSA, *SHOX2* methylation and *SEPT9* at baseline and after treatment for 20 days *n* = 6) [155]. In a small cohort (*n* = 6), PCa patients positive at baseline for PSA, *SHOX2*, and *SEPT9* methylation were followed during treatment. Despite high inter-patient variability, all patients exhibited decreased levels of the three markers, with *SEPT9* methylation showing the most rapid decline (to 25% of baseline within 10 days), suggesting its potential as an early indicator of the therapeutic response.

Well-established PCa biomarkers such as *GSTP1* and *APC* have also been investigated in advanced disease. In plasma from CRPCa patients (MSP; CRPCa patients *n* = 47; age-matched HDs *n* = 10; young HDs *n* = 10 and female HDs *n* = 10) [156], both markers were significantly hypermethylated compared to age-matched HDs (*p* < 0.01) and were associated with poorer OS. Their combination with the PSA levels improved the OS risk stratification, highlighting the value of integrated biomarker models. Similarly, a three-DMG plasma panel (qMSRE; *AKR1B1*, *LDAH*, and *KLF8*) discriminated mCRPa responders (*n* = 17) from non-responders (*n* = 12) to various therapies with high efficiency (AUC values of 0.93, 0.77 and 0.98, respectively) [130]. Beyond targeted panel, new methodologies have emerged to monitor treatment resistance using cfDNA. Using qMSP-ddPCR, Peter et al. (2022) tracked anonymized DMCpGs in serial samples (PCa patients *n* = 46; 26/46 longitudinally) [157,158] and found specific hypermethylation events associated with disease progression during ARi therapy. However, combining the methylation score with PSA levels did not improve predictive performance compared to PSA levels alone. In contrast, unbiased methylome analyses (H450K BC) performed directly in plasma from PCa patients treated with abiraterone (PCa *n* = 33) identified 33 DMCpGs in 22 genes that discriminated responders from non-responders [159]. Strikingly, 21 of these CpGs were already altered at baseline, supporting their potential as early predictive biomarkers of resistance.

Longitudinal plasma methylomes (*n* = 9 mPCa patients, 48 samples across 19 months) [160] further demonstrated that most CpGs remained stable over time, but subsets of CpGs underwent dynamic changes during taxane or ARi treatment. Notably, CpG methylation frequencies tended to revert to baseline after treatment discontinuation, suggesting that dynamic cfDNA methylation profiling could serve as a real-time indicator of the therapeutic response and drug-induced epigenetic reprogramming.

## 5. Conclusions

**Figure 3:**
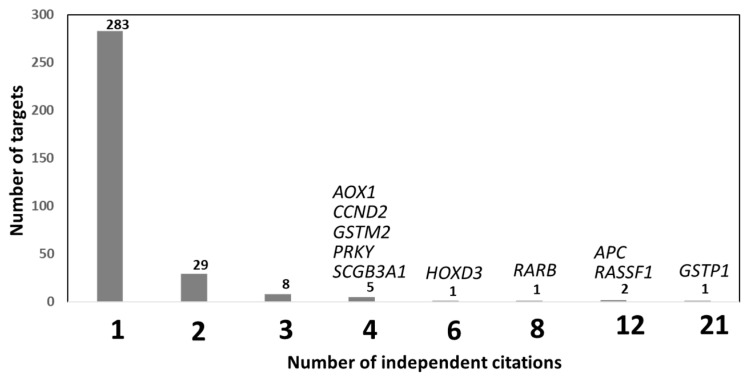
Classification of methylation PCa biomarkers in regard to independent citations.

As summarized in this review, the past decade has seen the identification of numerous DMRs with putative or validated roles in PCa diagnosis, prognosis, or prediction of the treatment response. Among the different DMCpGs discussed in this review (Supplementary Table S1), 274 different DMGs have been reported. Each methylation biomarker has been listed in Supplementary Table S1 with its acronym, full name, Cpg position when communicated, type of cohorts (tissues, liquid biopsies, public databases, and/or local cohorts) and its proposed clinical role. At first sight, this high number of markers for a cancer could be surprising. However, although all reasons are not fully understood, some hypothesis and observations have to be noted. *GSTP1* emerged as the most frequently DMG (21 references in this review), with its methylation consistently reported as informative for both the diagnosis and prognosis across different sample types (Figure 3). *RASSF1* and *APC* methylation, *RARB* methylation, and *HOXD3* methylation have also been, respectively, cited twelve, eight, and six times in different publications (Figure 3).

This finding strongly supports that these DMGs and specifically *GSTP1* methylation could be robust markers for PCa. Moreover, *GSTP1* methylation was one of the first methylation markers identified for PCa, probably due to its robustness. However, all authors did not propose *GSTP1* methylation in their panel. A few reasons could be proposed: (i) Most authors look for novelty in a publication strategy, and need to propose new biomarkers even if *GSTP1* methylation is found in their cohort. (ii) Many publications used the same methylome sets from public databases (TCGA and GEO) with bias toward confirming already published results obtained with the same data. However, when authors use independent cohorts, different methylation markers may emerge due to patient ethnicity, technical bias, or statistical analysis. (iii) The oldest studies dealing with methylation markers in PCa (2000–2005) focused on a specific gene, mostly for historical reasons of each laboratory. More recently, for the purposes of identifying independent markers and the best ones, more and more studies have used deep learning to test large methylomes and identify the best targets (generally hundreds of putative high-performance targets). This lead to the identification of very robust markers that are often different from the well-known markers (including *GSTP1* methylation). Moreover, although generating a similar list of putative biomarkers, due to the specificity of the process related to deep learning, a new run with the same set of data frequently classified these markers in a different order within the list. Since most authors now propose methylation panels frequently built with the first targets of their list, deep learning tends to increase the diversity of biomarkers. iii) Similar to many other cancers, PCa is not a homogeneous disease. Some PCa subtypes (such as NE PCa and CRPCa) may be associated with specific methylation events, increasing the quantity of methylation markers proposed for PCa identification.

**Figure 4:**
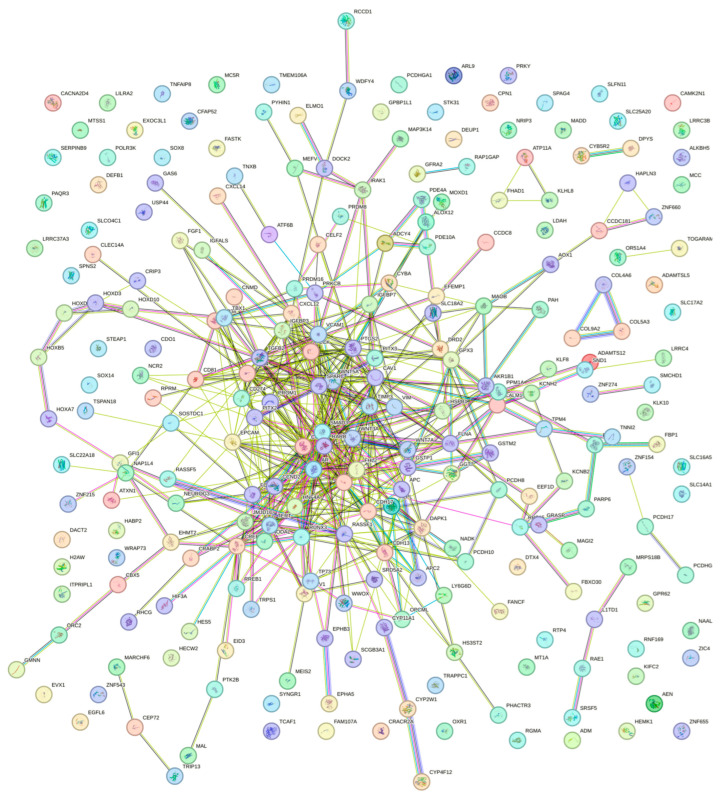
Interactome pathways of the 274 PCa-related DMGs listed in this review (String-db.org). Known interactions—blue (curated databases) and pink (experimentally shown); predicted interactions—green (gene neighborhood), red (gene fusions), blue (co-occurrence), light green (text mining), black (co-expression), and light blue (homology).

STRING pathway analysis indicated that most of DMGs are involved in the regulation of transcription and proliferation (Figure 4 and Table 1), supporting the notion that DNA methylation modifications may drive PCa carcinogenesis through the epigenetic regulation of cancer-relevant genes.

Since some *loci* could be methylated during carcinogenesis independently of their function, a specific CpG methylation event could be relevant for cancer diagnosis without affecting gene expression. However, methylation-mediated invalidations of genes involved in cell cycle control, apoptosis, or other specific pathways (metabolism, drug resistance, etc.) are well-described in many cancers. Although these methylation events are beneficial for tumor cells, they could also very efficiently be used for determining the prognosis and predicting the treatment response. Depending on the question tested by the authors (survival, metastasis, grade, response to ARis, radioresistance, etc.) different methylation targets could be obtained within a single cohort. As a large diversity of these methylation events affect the processes cited above, all of them could be helpful for clinicians.

Methylation-related biomarkers could be used in solid biopsies but their power would considerably help clinicians if they could be applicable in non-invasive, liquid biopsies (Figure 1). However, this review highlights the recurrent difficulty of transferring efficient biomarkers from tissues to liquid biopsies, where reduced sensitivity and specificity are frequently observed. A drop in sensitivity or efficiency is frequently observed. cftDNA purification and especially bisulfite conversion are associated with a quite poor yield. Indeed, cftDNA concentrations are often low or even very low (<1 ng/µL). Consequently, the thresholds for signal detection using classical techniques are frequently unreachable, except for metastasis patients who generally present high cftDNA concentrations. This point is, until now, a severe limitation for the routine and efficient use of methylation in clinics. Moreover, since tumors are heterogeneous, it might be possible that tumor cells that die first and therefore release cftDNA are not fully representative of the global tumor mass. In that case, most high-performance methylation biomarkers previously designed in solid tissues (local cohorts but mainly with public databases) may be weaker in liquid biopsies.

In response to these difficulties, several groups have shifted effort toward directly identifying novel DMGs in liquid biopsies. This raises a central question: How many biomarkers, and which technical approaches, are needed to provide clinicians with reliable diagnostic and prognostic information? Based on a deep learning approach built on methylomes from tissues, we recently published that for colon cancer, in theory, no gain in AUC performance would be obtained with a seventh methylation marker in a panel [161]. Indeed, only few increases in AUC were obtained from the third biomarker.

Although methylation assays remain more expensive than PSA quantification, it appears unlikely that short biomarker panels alone will adequately address all clinical questions (diagnosis, prognosis, and treatment resistance) or capture population-specific variability. Due to the costs and low amounts of materials, very large panels associated with PCR (as seen above) show excellent accuracy but remain impractical for routine use.

Bisulfite sequencing offers the advantage of a whole-genome technique but is not really realistic in clinics.

**Figure 5:**
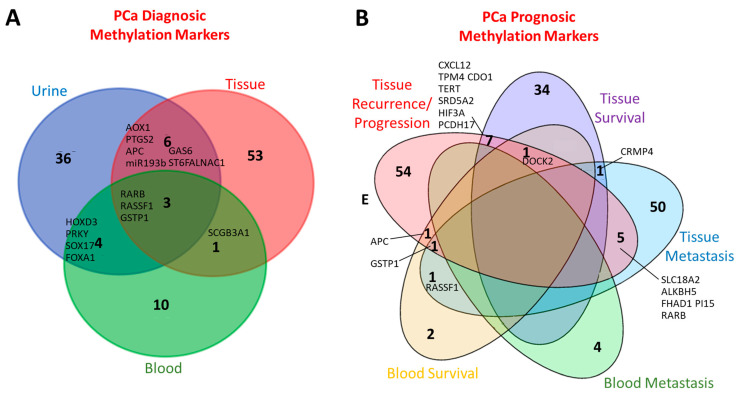
Venn diagram of genes hypermethylated in PCa. (**A**) Methylated genes identified for diagnosis in urine, tissues, and blood. (**B**) Methylated genes identified for the prognosis of recurrence and progression, survival, and metastasis in tissues and blood.

Among the 274 DMGs complied in this review, it remains difficult to choose the best ones for clinical applications. By analyzing the hypermethylated genes associated with PCa diagnosis using a Venn diagram (Figure 5A), it appears that only three genes (*RARB*, *RASSF1* and *GSTP1*) were identified in tissues, urine, and blood suggesting that these targets are robust and may be included in most panels. Six, four, and one additional targets were, respectively, found in tissues and urine, urine and blood, and tissues and blood. Concerning the hypermethylated genes associated with prognosis (Figure 5B), most of hypermethylated genes identified for survival, recurrence/progression, or metastasis were found in a single category. Five of them were found in tissues for both the metastasis risk and recurrence/progression, and seven were found in tissues for both survival and recurrence/progression. As discussed above, very few targets, including *GSTP1* and *APC*, were found simultaneously in tissues and liquid biopsies.

However, we could hypothesis that those presenting good performances in liquid biopsies may appear the most promising until now for clinical application. Table 2 summarizes the panels used in liquid biopsies and the associated sensitivity and specificity values. Although some of them seem to have good performance, the lack of validation by an independent group and sometimes the small size are limitations.

The recent emergence of sequencing technologies, particularly nanopore-based approaches, may provide a paradigm shift by enabling cost-effective, patient-specific whole-genome methylome profiling. This technology does not require bisulfite conversion, which increases its sensitivity compared to most classical techniques and facilitates the analysis of reads. Since samples could be barcoded in libraries, this would drop the individual costs and seems compatible with clinics. Moreover, as genetic information (mutational status) would be obtained simultaneously, some specific molecular tests would be cancelled.

Such strategies would remove the need for predefined panels and instead rely on comprehensive methylation signatures benchmarked against reference methylomes. Importantly, studies suggest that incorporating hundreds of CpG targets can yield AUC values above 0.9 in liquid biopsies, significantly improving diagnostic and prognostic performance. The major remaining challenge is logistical, as widespread clinical implementation would require robust bioinformatics infrastructure to support analysis and interpretation and yet nanopore technology remains associated with a quite high error rate in sequencing.

## Figures and Tables

**Table 1:** Gene Ontology of DMGs related to PCa (String-db.org).

Function	Fdr
Molecular function	
Transcription factor binding	0.0004
RNA polymerase II cis-regulatory region sequence-specific DNA binding	0.0007
DNA-binding transcriptional factor	0.002
RNA polymerase II transcription regulatory region sequence-specific DNA binding	0.002
Biological process	
Regulation of epithelial cell proliferation	2 × 10^−7^
Response to endogenous stimulus	8.8 × 10^−10^
Epithelial cell differentiation	2.8 × 10^−6^

**Table 2:** Comparison of panel performances for PCa diagnosis in liquid biopsies.

Biomarker	Methylation Panel	Liquid Biopsy	Cohort	Technique	Specificity	Sensitivity	Reference
Diagnosis	GSTP1, RASSF1, RASSF2	Plasma	PCa *n* = 13; HG neoplasia *n* = 3; BPH *n* = 20; ASAP *n* = 3; HD *n* = 15	MSP	83%	8%	[28]
Diagnosis	GSTP1, HOXD3	Urine	PCa *n* = 408 and BPH *n* = 182	MSP	97%	57%	[37]
Diagnosis	miR34c, miR193b	Urine	PCa *n* = 87; HD *n* = 32	MSP	92%	95%	[38]
Diagnosis	miR193b	Urine	PCa *n* = 95; non-urological cancer *n* = 29; HD *n* = 17	MBD capture-PCR	96%	92%	[40]
Diagnosis	AOX1rc, APC2, CXCL14, EPHX3, KIFC2, GFRA2, GSTP1, NEUROG3, NODAL, RASSF5, HEMK1, HOXA7, HOXB5, HOXD3a, HOXD3b, HOXD10, MOXD1	Urine	PCa *n* = 42; controls *n* = 50	MSP	70%	90%	[41]
Diagnosis	AKR1B1HES5, CHST11, GAS6, GRASP, ITPRIPL1, KCNB2, MAX.chr3.6187, AX.chr3.8.28, SCOL3A1, SERPIN B9, ST6GALNAC2, WNT3A, ZNF655	Urine	PCa *n* = 24; HD *n* = 24	bisulfite sequencing	100%	59%	[42]
Diagnosis	APC, FOXA1, GSTP1, HOXD3, RARB2, RASSF1A, SEPT9, SOX17	Plasma	PCa *n* = 121	MSP	72%	72%	[46]

## Supplementary Materials

The following supporting information can be downloaded at: https://www.mdpi.com/article/10.3390/biom15091334/s1, Table S1: List of 274 Differentially Methylated Genes (DMGs) and Asso-ciated Biomarkers: Acronyms, Full Names, CpG Positions, Cohorts, and Proposed Clinical Roles.
