# Supplementary material for: The Years 2015–2025 as a Prospective Decade for the Identification of Specific Methylation Biomarkers of Prostate Cancer
Source: Biomolecules. 2025 Sep 18;15(9):1334. doi: 10.3390/biom15091334 (PMC12467249; doi:10.3390/biom15091334)
Supplement: Supplementary file 1 [file biomolecules-15-01334-s001.zip › biomolecules-3775665-supplementary.pdf]

ALIAS	FULL NAME
AC005786.5	
AC006116.21	
AC074091.13	
AC087499.10	
AC090954.1	
AC091801.1	
AC097532.1	
AC116562.1	
ADAMTS12	ADAM Metallopeptidase With Thrombospondin Type 1 Motif 12
ADAMTSL5	ADAM Metallopeptidase With Thrombospondin Type 1 Motif 5
ADCY4	Adenylate Cyclase 4
ADD3-AS1	ADD3 Antisense RNA 1
ADM	Adrenomedullin
AEN	Apoptosis Enhancing Nuclease
AF228730.1	
AKR1B1	Aldo-Keto Reductase Family 1 Member B
ALDH1L1-AS2	ALDH1L1 Antisense RNA 2
ALKBH5	AlkB Homolog 5, RNA Demethylase
ALOX12	Arachidonate 12-Lipoxygenase, 12S Type
ALPL	Alkaline Phosphatase, Biomineralization Associated
AOX1	Aldehyde Oxidase 1
AP003498.1	
APC	APC Regulator Of WNT Signaling Pathway
APC2	APC Regulator Of WNT Signaling Pathway 2
AR	Androgen Receptor
ARHGAP42P1	ARHGAP42 Pseudogene 1
ARL9	ARF Like GTPase 9
ATF6B	Activating Transcription Factor 6 Beta
ATP11A	ATPase Phospholipid Transporting 11A
ATXN1	Ataxin 1
B3GNLT1	Beta-1,3-Glucuronyltransferase 1
BHLHB9 / GAS3	G Protein-Coupled Receptor Associated Sorting Protein Family Member 3

BMS1P4	BMS1 Pseudogene 4
CACNA1G-AS1	CACNA1G Antisense RNA 1
CACNA2D4	Calcium Voltage-Gated Channel Auxiliary Subunit Alpha2delta 4
CALM1	Calmodulin 1
CAMK2N1	Calcium/Calmodulin Dependent Protein Kinase II Inhibitor 1
CAV1	Caveolin 1
CBX5	Chromobox 5
CCDC181	Coiled-Coil Domain Containing 181
CCDC8	Coiled-Coil Domain Containing 8
CCK	Cholecystokinin
CCND1	Cyclin D1
CCND2	Cyclin D2
CD34	CD34 Molecule
CD38	CD38 Molecule
CD81	CD81 Molecule
CDC2/CDK1	Cyclin Dependent Kinase 1
CDH1	Cadherin 1
CDH13	Cadherin 13
CDH17	Cadherin 17
CDO1	Cysteine Dioxygenase Type 1
CEP72	Centrosomal Protein 72
chr1:146522288-146522555	
chr1:2772126-27726	
Chr10:23	
Chr12:34	
chr14:104596580-104598145	
chr16:33038921-33040607	
chr18:15165109-15165342 (CpG island name)	
chr19:54775040-54775040	
chr3:129855195-129855560	
Chr4:13	
Chr6:104	
Chr7:154	
chr7:63634549-63635889	
chr7:92672789-92673016	

chr8:143645072-143645673 (CpG island name)	
CHST11	Carbohydrate Sulfotransferase 11
CLEC14A	C-Type Lectin Domain Containing 14A
CNMD	Chondromodulin
COL4A6	Collagen Type IV Alpha 6 Chain
COL5A3	Collagen Type V Alpha 3 Chain
COL9A2	Collagen Type IX Alpha 2 Chain
CPN1	Carboxypeptidase N Subunit 1
CRABP2	Cellular Retinoic Acid Binding Protein 2
CRACR2A	Calcium Release Activated Channel Regulator 2A
CREBBP	CREB Binding Protein
CRIP3	Cysteine Rich Protein 3
CRMP4/DPYLS3	Collapsin Response Mediator Protein 4
CTB-83J4.1	
CTD-2555A7.2	
CUGBP2	CUG Triplet Repeat, RNA-Binding Protein 2
CXCL12	C-X-C Motif Chemokine Ligand 12
CXCL14	C-X-C Motif Chemokine Ligand 14
CYB5R2	Cytochrome B5 Reductase 2
CYBA	Cytochrome B-245 Alpha Chain
CYP11A1	Cytochrome P450 Family 11 Subfamily A Member 1
CYP2W1	Cytochrome P450 Family 2 Subfamily W Member 1
CYP4F12	Cytochrome P450 Family 4 Subfamily F Member 12
DACT2	Dishevelled Binding Antagonist Of Beta Catenin 2
DAPK1	Death Associated Protein Kinase 1
DEFB1	Defensin Beta 1
DEUP1	Deuterosome Assembly Protein 1
DOCK2	Dedicator Of Cytokinesis 2
DPYS	Dihydropyrimidinase
DRD2	Dopamine Receptor D2
DTX4	Deltex E3 Ubiquitin Ligase 4
EEF1D	Eukaryotic Translation Elongation Factor 1 Delta
EFEMP1	EGF Containing Fibulin Extracellular Matrix Protein 1
EGFL6	EGF Like Domain Multiple 6
EHMT2	Euchromatic Histone Lysine Methyltransferase 2
EID3	EP300 Interacting Inhibitor Of Differentiation 3
ELMO1	Engulfment And Cell Motility 1
EPCAM	Epithelial Cell Adhesion Molecule
EPHA5	EPH Receptor A5
EPHB3	EPH Receptor B3

EPHB3	EPH Receptor B3
ETV1	ETS Variant Transcription Factor 1
EVX1	Even-Skipped Homeobox 1
EXOC3L1	Exocyst Complex Component 3 Like 1
F11-AS1	F11 Antisense RNA 1
FAM107A	Family With Sequence Similarity 107 Member A
FAM179A	Family With Sequence Similarity 179, Member A
FAM66A	Family With Sequence Similarity 66 Member A
FANCF	FA Complementation Group F
FASTK	Fas Activated Serine/Threonine Kinase
FBP1	Fructose-Bisphosphatase 1
FBXO30	F-Box Protein 30
FGF1	Fibroblast Growth Factor 1
FGF14-AS2	FGF14 Antisense RNA2
FHAD1	Forkhead Associated Phosphopeptide Binding Domain 1
FHL2	Four And A Half LIM Domains 2
FLNA	Filamin A
FLRT2	Fibronectin Leucine Rich Transmembrane Protein 2
FOXA1	Forkhead Box A1
FOXP1	Forkhead Box P1
GABRQ	Gamma-Aminobutyric Acid Type A Receptor Subunit Theta
GADD45A	Growth Arrest And DNA Damage Inducible Alpha
GARIN1A	Golgi Associated RAB2 Interactor 1A
GAS6	Growth Arrest Specific 6
GFI1	Growth Factor Independent 1 Transcriptional Repressor
GFRA2	GDNF Family Receptor Alpha 2
GGT7	Gamma-Glutamyltransferase 7
GMNN	Geminin DNA Replication Inhibitor
GPBP1L1	GC-Rich Promoter Binding Protein 1 Like 1
GPR62	G Protein-Coupled Receptor 62
GPX3	Glutathione Peroxidase 3
GRASP	GRP1 (General Receptor For Phosphoinositides 1)-Associated Scaffold Protein
GSTM2	Glutathione S-Transferase Mu 2

GSTP1	Glutathione S-Transferase Pi 1
GTSE1-AS1	GTSE1 Antisense RNA 1
HABP2	Hyaluronan Binding Protein 2
HAPLN3	Hyaluronan And Proteoglycan Link Protein 3
HECW2	HECT, C2 And WW Domain Containing E3 Ubiquitin Protein Ligase 2
HEMK1	HemK Methyltransferase Family Member 1
HES5	Hes Family BHLH Transcription Factor 5
HIF3A	Hypoxia Inducible Factor 3 Subunit Alpha
HIST3H2A	Histone H2A Type 3
HNF4A	Hepatocyte Nuclear Factor 4 Alpha
HOXA7	Homeobox A7
HOXB5	Homeobox B5
HOXD10	Homeobox D10
HOXD3	Homeobox D3
HOXD9	Homeobox D9
HS3ST2	Heparan Sulfate-Glucosamine 3-Sulfotransferase 2
HSPB1	Heat Shock Protein Family B (Small) Member 1
IGFALS	Insulin Like Growth Factor Binding Protein Acid Labile Subunit
IGFBP3	Insulin Like Growth Factor Binding Protein 3
IGFBP7	Insulin Like Growth Factor Binding Protein 7
IRAK1	Interleukin 1 Receptor Associated Kinase 1
ITPRIPL1	ITPRIP Like 1
JMJD1C	Jumonji Domain Containing 1C
KCNB2	Potassium Voltage-Gated Channel Subfamily B Member 2
KCNH2	Potassium Voltage-Gated Channel Subfamily H Member 2

KIFC2	Kinesin Family Member C2
KLF8	KLF Transcription Factor 8
KLHL8	Kelch Like Family Member 8
KLK10	Kallikrein Related Peptidase 10
LDAH	Lipid Droplet Associated Hydrolase
LILRA2	Leukocyte Immunoglobulin Like Receptor A2
LINC00354	Long Intergenic Non-Protein Coding RNA 354
LINC00403/SOX1-OT	Long Intergenic Non-Protein Coding RNA 403
LINC00506	Long Intergenic Non-Protein Coding RNA 506
LINC01091	Long Intergenic Non-Protein Coding RNA 1091
LINC01122	Long Intergenic Non-Protein Coding RNA 1122
LINE1	Line-1
LOC100289511	
LOC643387	
LRRC37A3	Leucine Rich Repeat Containing 37 Member A3
LRRC3B	Leucine Rich Repeat Containing 3B
LRRC4	Leucine Rich Repeat Containing 4
LY6G6D	Lymphocyte Antigen 6 Family Member G6D
MADD	MAP Kinase Activating Death Domain
MAGI2	Membrane Associated Guanylate Kinase, WW And PDZ Domain Containing 2
MAL	Mal, T Cell Differentiation Protein
MAOB	Monoamine Oxidase B
MAP3K14	Mitogen-Activated Protein Kinase Kinase Kinase 14
MARCH6	Membrane Associated Ring-CH-Type Finger 6
MAX.chr3.6187	
MAX.chr3.8028	
MC5R	Melanocortin 5 Receptor
MCC	MCC Regulator Of WNT Signaling Pathway
MEFV	MEFV Innate Immunity Regulator, Pyrin
MEG3	Maternally Expressed 3
MEIS2	Meis Homeobox 2
miR-1258	MicroRNA 1258
miR129-2	MicroRNA 129-2
miR-152	MicroRNA 152
miR193b	MicroRNA 193-b
miR-34b/C	MicroRNA 34b-c
miR-663a	MicroRNA 663a
miR9-3HG	MIR9-3 Host Gene
MOXD1	Monooxygenase DBH Like 1
MRPS18B	Mitochondrial Ribosomal Protein S18B

MSC-AS1	MSC Antisense RNA 1
MT1A	Metallothionein 1A
MTSS1	MTSS I-BAR Domain Containing 1
MYCL2	MYCL Proto-Oncogene 2
NAALAD2	N-Acetylated Alpha-Linked Acidic Dipeptidase 2
NADK	NAD Kinase
NAP1L4	Nucleosome Assembly Protein 1 Like 4
NCR2	Natural Cytotoxicity Triggering Receptor 2
NCS1/FREQ	Neuronal Calcium Sensor 1
NEUROG3	Neurogenin 3
NNT-AS1	NNT Antisense RNA 1
NODAL	Nodal Growth Differentiation Factor
NRIP3	Nuclear Receptor Interacting Protein 3
OPCML	Opioid Binding Protein/Cell Adhesion Molecule Like
OR51A4	Olfactory Receptor Family 51 Subfamily A Member 4
ORC2L	Origin Recognition Complex Subunit 2
OXR1	Oxidation Resistance 1
PAH	Phenylalanine Hydroxylase
PAQR3	Progestin And AdipoQ Receptor Family Member 3
PARP6	Poly(ADP-Ribose) Polymerase Family Member 6
PCDH10	Protocadherin 10
PCDH17	Protocadherin 17
PCDH8	Protocadherin 8
PCDHGA1	Protocadherin Gamma Subfamily A, 1
PCDHGC4	Protocadherin Gamma Subfamily C, 4
PDE10A	Phosphodiesterase 10A
PDE4A	Phosphodiesterase 4A
PD-L1	Programmed Cell Death Ligand 1
PFKP	Phosphofructokinase, Platelet
PGM5	Phosphoglucomutase 5
PHACTR3	Phosphatase And Actin Regulator 3
PI15	Peptidase Inhibitor 15
PITX2	Paired Like Homeodomain 2
PITX3	Paired Like Homeodomain 3
PLAAT3/PLA2G16	Phospholipase A And Acyltransferase 3
POLR3K	RNA Polymerase III Subunit K
PPM1A	Protein Phosphatase, Mg2+/Mn2+ Dependent 1A
PPM1K-DT	PPM1K Divergent Transcript
PRDM16	PR/SET Domain 16
PRDM8	PR/SET Domain 8
PRKCB	Protein Kinase C Beta

PRKY	Protein Kinase Y-Linked (Pseudogene)
PROM1	Prominin 1
PTGS2	Prostaglandin-Endoperoxide Synthase 2
PTK2B	Protein Tyrosine Kinase 2 Beta
PVT1	Pvt1 Oncogene
PYHIN1	Pyrin And HIN Domain Family Member 1
RAE1	Ribonucleic Acid Export 1
RAP1GAP	RAP1 GTPase Activating Protein
RARB	Retinoic Acid Receptor Beta
RASSF1	Ras Association Domain Family Member 1
RASSF5	Ras Association Domain Family Member 5
RCCD1	RCC1 Domain Containing 1
RGMA	Repulsive Guidance Molecule BMP Co-Receptor A
RHCG	Rh Family C Glycoprotein
RNF169	Ring Finger Protein 169
RP11-201M22.1	
RP11-379F4.4;MFSD1	
RP11-423O2.7	
RP11-74E22.8	
RP11–760D2.1	
RP5-1159O4.1	
RPL35AP31	Ribosomal Protein L35a Pseudogene 31
RPS15	Ribosomal Protein S15
RREB1	Ras Responsive Element Binding Protein 1
RTP4	Receptor Transporter Protein 4

RUNX3	RUNX Family Transcription Factor 3
SCGB3A1	Secretoglobin Family 3A Member 1
SCOL3A1	
SDK1-AS1	SDK1 Antisense RNA 1
SERPINB9	Serpin Family B Member 9
SETP9	SET Pseudogene 9
SFRS5	Serine And Arginine Rich Splicing Factor 5
SLC14A1	Solute Carrier Family 14 Member 1 (Kidd Blood Group)
SLC16A5	Solute Carrier Family 16 Member 5
SLC17A2	Solute Carrier Family 17 Member 2
SLC18A2	Solute Carrier Family 18 Member A2
SLC22A18	Solute Carrier Family 22 Member 18
SLC25A20	Solute Carrier Family 25 Member 20
SLCO4C1	Solute Carrier Organic Anion Transporter Family Member 4C1
SLFN11	Schlafen Family Member 11
SMAD3	SMAD Family Member 3
SMCHD1	Structural Maintenance Of Chromosomes Flexible Hinge Domain Containing 1
SND1	Staphylococcal Nuclease And Tudor Domain Containing 1
SNORA10	Small Nucleolar RNA, H/ACA Box 10
SOSTDC1	Sclerostin Domain Containing 1
SOX11	SRY-Box Transcription Factor 11
SOX14	SRY-Box Transcription Factor 14
SOX17	SRY-Box Transcription Factor 17
SOX8	SRY-Box Transcription Factor 8
SPAG4	Sperm Associated Antigen 4
SPARC	Secreted Protein Acidic And Cysteine Rich
SPNS2	SPNS Lysolipid Transporter 2, Sphingosine-1-Phosphate
SRD5A2	Steroid 5 Alpha-Reductase 2
ST6FALNAC1	Alpha-N-acetylgalactosaminide alpha-2,6-sialyltransferase 1
STEAP1	STEAP Family Member 1
STK31	Serine/Threonine Kinase 31
SYNGR1	Synaptogyrin 1
TBX1	T-Box Transcription Factor 1
TCAF1	TRPM8 Channel Associated Factor 1
TGFB2	Transforming Growth Factor Beta 2
TERT	Telomerase Reverse Transcriptase

TIG1/RARRES1	Tazarotene-Induced Gene 1 Protein
TIMP3	TIMP Metallopeptidase Inhibitor 3
TMEM106A	Transmembrane Protein 106A
TNFAIP8	TNF Alpha Induced Protein 8
TNFRF10c	TNF Receptor Superfamily Member 10c
TNNI2	Troponin I2, Fast Skeletal Type
TNXB	Tenascin XB
TP73	Tumor Protein P73
TPM4	Tropomyosin 4
TRAPPC1	Trafficking Protein Particle Complex Subunit 1
TRIP13	Thyroid Hormone Receptor Interactor 13
TRPS1	Transcriptional Repressor GATA Binding 1
TSPAN18	Tetraspanin 18
USP44	Ubiquitin Specific Peptidase 44
VCAM1	Vascular Cell Adhesion Molecule 1
VIM	Vimentin
VX1	
WDFY4	WDFY Family Member 4
WDR16	WD Repeat-Containing Protein 16
WDR8	WD Repeat Domain 8
WNT3A	Wnt Family Member 3A
WNT5A	Wnt Family Member 5A
WNT7A	Wnt Family Member 7A
WWC2-AS2	WWC2 Antisense RNA 2
WWOX	WW Domain Containing Oxidoreductase
ZIC4	Zic Family Member 4
ZNF154	Zinc Finger Protein 154
ZNF215	Zinc Finger Protein 215
ZNF274	Zinc Finger Protein 274
ZNF543	*Zinc Finger Protein 543*
ZNF655	Zinc Finger Protein 665
ZNF660	Zinc Finger Protein 660

ROLE		COHORTS	
PROGNOSIS	OS	TISSUES	PUBLIC DATABASE
PROGNOSIS	OS	TISSUES	PUBLIC DATABASE
PREDICTIVE	RECURRENCE	TISSUES	LOCAL COHORT
PREDICTIVE	METASTASIS	tissue	LOCAL COHORT
PREDICTIVE	METASTASIS	tissue	LOCAL COHORT
PROGNOSIS	OS	TISSUES	PUBLIC DATABASE
PREDICTIVE	METASTASIS	tissue	LOCAL COHORT
PREDICTIVE	METASTASIS	tissue	LOCAL COHORT
PREDICTIVE	RECURRENCE	TISSUES	PUBLIC DATABASE
PROGNOSIS	METASTASIS	TISSUES	LOCAL COHORT
DIAGNOSIS		URINE	LOCAL COHORT
PREDICTIVE	METASTASIS	tissue	LOCAL COHORT
PROGNOSIS	METASTASIS	TISSUES	LOCAL COHORT
PROGNOSIS	METASTASIS	TISSUES	LOCAL COHORT
PREDICTIVE	METASTASIS	tissue	LOCAL COHORT
PREDICTIVE	RESPONSE TO TREATMENT	BLOOD	LOCAL COHORT
DIAGNOSIS		TISSUES	PUBLIC DATABASE
PREDICTIVE	METASTASIS	tissue	LOCAL COHORT
PROGNOSIS	RECURRENCE	TISSUES	LOCAL COHORT
PROGNOSIS	METASTASIS	TISSUES	LOCAL COHORT
PROGNOSIS	RECURRENCE	TISSUES	LOCAL COHORT
DIAGNOSIS		TISSUES	PUBLIC DATABASE
PROGNOSIS	METASTASIS	TISSUES	LOCAL COHORT
DIAGNOSIS		TISSUES	LOCAL COHORT/PUB
DIAGNOSIS		TISSUES	LOCAL COHORT
DIAGNOSIS		URINE	LOCAL COHORT
PREDICTIVE	METASTASIS	tissue	LOCAL COHORT
PROGNOSIS	RECURRENCE	TISSUES	LOCAL COHORT
PROGNOSIS	RECURRENCE	TISSUES	LOCAL COHORT
DIAGNOSIS		TISSUES	LOCAL COHORT
DIAGNOSIS	HIGH-RISK	URINE	LOCAL COHORT
PROGNOSIS	RECURRENCE GRADE	TISSUES	LOCAL COHORT
PROGNOSIS	OS	BLOOD	LOCAL COHORT
DIAGNOSIS		TISSUES	LOCAL COHORT
PROGNOSIS	RECURRENCE	TISSUES	LOCAL COHORT
PROGNOSIS	GRADE	TISSUES	LOCAL COHORT
PROGNOSIS	GRADE	TISSUES	LOCAL COHORT
DIAGNOSIS		TISSUES	LOCAL COHORT
DIAGNOSIS		URINE	LOCAL COHORT
DIAGNOSIS		URINE	LOCAL COHORT
PROGNOSIS	BONE METASTASIS	TISSUES	LOCAL COHORT
PROGNOSIS	METASTASIS	TISSUES	LOCAL COHORT
DIAGNOSIS		TISSUES	LOCAL COHORT
PREDICTIVE	PROGRESSION	TISSUES	LOCAL COHORT
PROGNOSIS	RECURRENCE	TISSUES	LOCAL COHORT
		TISSUES	PUBLIC DATABASE
DIAGNOSIS		TISSUES	LOCAL COHORT
PREDICTIVE	SURVIVAL	TISSUES	LOCAL COHORT

PROGNOSIS		TISSUES	PUBLIC DATABASE
PROGNOSIS		TISSUES	PUBLIC DATABASE
PREDICTIVE	RECURRENCE	TISSUES	LOCAL COHORT
PREDICTIVE	SURVIVAL	TISSUES	LOCAL COHORT
PREDICTIVE	PFS	TISSUES	LOCAL COHORT/PUB
		TISSUES	PUBLIC DATABASE
DIAGNOSIS		TISSUES	LOCAL COHORT
DIAGNOSIS		TISSUES	LOCAL COHORT
DIAGNOSIS		TISSUES	PUBLIC DATABASE
DIAGNOSIS		TISSUES	LOCAL COHORT
DIAGNOSIS		TISSUES	PUBLIC DATABASE
PREDICTIVE	RECURRENCE	TISSUES	PUBLIC DATABASE
PROGNOSIS	METASTASIS	TISSUES	LOCAL COHORT
DIAGNOSIS		TISSUES	LOCAL COHORT
PROGNOSIS	OS	TISSUES	LOCAL COHORT
DIAGNOSIS		TISSUES	LOCAL COHORT
DIAGNOSIS		TISSUES	PUBLIC DATABASE
PREDICTIVE	SURVIVAL	TISSUES	LOCAL COHORT
PREDICTIVE	RECURRENCE	TISSUES	PUBLIC DATABASE
PROGNOSIS	METASTASIS	TISSUES	LOCAL COHORT
PROGNOSIS	METASTASIS	TISSUES	LOCAL COHORT
DIAGNOSIS		URINE	LOCAL COHORT
DIAGNOSIS		BLOOD	LOCAL COHORT
PREDICTIVE	SURVIVAL	TISSUES	LOCAL COHORT
PREDICTIVE	SURVIVAL	TISSUES	LOCAL COHORT
PROGNOSIS	RECURRENCE PFS	TISSUES	LOCAL COHORT/PUBI
PREDICTIVE	PROGRESSION	TISSUES	LOCAL COHORT
PREDICTIVE	METASTASIS	tissue	LOCAL COHORT
PREDICTIVE	METASTASIS	tissue	LOCAL COHORT
DIAGNOSIS		TISSUES	PUBLIC DATABASE
DIAGNOSIS		TISSUES	PUBLIC DATABASE
PREDICTIVE	METASTASIS	tissue	LOCAL COHORT
PREDICTIVE	METASTASIS	tissue	LOCAL COHORT
PREDICTIVE	METASTASIS	tissue	LOCAL COHORT
PREDICTIVE	METASTASIS	tissue	LOCAL COHORT
PREDICTIVE	METASTASIS	tissue	LOCAL COHORT
DIAGNOSIS		TISSUES	PUBLIC DATABASE
DIAGNOSIS		TISSUES	PUBLIC DATABASE
DIAGNOSIS		TISSUES	PUBLIC DATABASE
PREDICTIVE	METASTASIS	tissue	LOCAL COHORT
PREDICTIVE	METASTASIS	tissue	LOCAL COHORT

PREDICTIVE	METASTASIS	tissue	LOCAL COHORT
PREDICTIVE	RECURRENCE	BLOOD	LOCAL COHORT
PREDICTIVE	METASTASIS	BLOOD	LOCAL COHORT
DIAGNOSIS		URINE	LOCAL COHORT
DIAGNOSIS		TISSUES	LOCAL COHORT
PREDICTIVE	SURVIVAL	TISSUES	LOCAL COHORT
DIAGNOSIS		TISSUES	PUBLIC DATABASE
PROGNOSIS	PFS	TISSUES	LOCAL COHORT
PROGNOSIS	METASTASIS	TISSUES	LOCAL COHORT
PROGNOSIS	METASTASIS	TISSUES	LOCAL COHORT
PROGNOSIS	METASTASIS	TISSUES	LOCAL COHORT
PROGNOSIS		BLOOD	LOCAL COHORT
PREDICTIVE	SURVIVAL	TISSUES	LOCAL COHORT
		TISSUES	PUBLIC DATABASE
PROGNOSIS	RECURRENCE	TISSUES	LOCAL COHORT
PROGNOSIS	RECURRENCE	TISSUES	LOCAL COHORT
PREDICTIVE	PROGRESSION	TISSUES	
PROGNOSIS	PFS	TISSUES	LOCAL COHORT
PROGNOSIS	METASTASIS	TISSUES	LOCAL COHORT
PROGNOSIS	OS	TISSUES	PUBLIC DATABASE
PROGNOSIS	OS	TISSUES	PUBLIC DATABASE
PREDICTIVE	METASTASIS	BLOOD	LOCAL COHORT
PROGNOSIS	RECURRENCE PFS	TISSUES	LOCAL COHORT/PUBI
DIAGNOSIS		URINE	LOCAL COHORT
DIAGNOSIS		BLOOD	LOCAL COHORT
DIAGNOSIS		TISSUES	PUBLIC DATABASE
PROGNOSIS	RECURRENCE	BLOOD	LOCAL COHORT
PREDICTIVE	RECURRENCE	TISSUES	PUBLIC DATABASE
DIAGNOSIS		TISSUES	LOCAL COHORT
PROGNOSIS	METASTASIS	TISSUES	LOCAL COHORT
PROGNOSIS	METASTASIS	BLOOD	
DIAGNOSIS		TISSUES	PUBLIC DATABASE
DIAGNOSIS		TISSUES	LOCAL COHORT
PREDICTIVE	SURVIVAL	TISSUES	LOCAL COHORT
PROGNOSIS	PFS	BLOOD	LOCAL COHORT
PROGNOSIS	RECURRENCE PFS	TISSUES	LOCAL COHORT/PUBI
PROGNOSIS	OS	TISSUES	LOCAL COHORT
DIAGNOSIS		BLOOD	LOCAL COHORT
DIAGNOSIS		TISSUES	LOCAL COHORT
PREDICTIVE	PROGRESSION	TISSUES	LOCAL COHORT
DIAGNOSIS		TISSUES	PUBLIC DATABASE
DIAGNOSIS		TISSUES	LOCAL COHORT
PREDICTIVE	PROGRESSION	TISSUES	LOCAL COHORT
PREDICTIVE	RECURRENCE	TISSUES	PUBLIC DATABASE
PREDICTIVE	METASTASIS	tissue	LOCAL COHORT
PROGNOSIS	PFS	TISSUES	PUBLIC DATABASE
PREDICTIVE	SURVIVAL	TISSUES	LOCAL COHORT

DIAGNOSIS		URINE	LOCAL COHORT
PREDICTIVE	RESISTANCE	TISSUES	LOCAL COHORT
DIAGNOSIS		TISSUES	LOCAL COHORT
DIAGNOSIS		TISSUES	PUBLIC DATABASE
PROGNOSIS		TISSUES	PUBLIC DATABASE
DIAGNOSIS		TISSUES	PUBLIC DATABASE
PROGNOSIS	METASTASIS	TISSUES	LOCAL COHORT
PREDICTIVE	METASTASIS	tissue	LOCAL COHORT
PREDICTIVE	PROGRESSION	TISSUES	LOCAL COHORT
PREDICTIVE	PROGRESSION	TISSUES	LOCAL COHORT
DIAGNOSIS		TISSUES	PUBLIC DATABASE
PROGNOSIS	PFS	BLOOD	LOCAL COHORT
DIAGNOSIS		TISSUES	LOCAL COHORT
PROGNOSIS	OS	TISSUES	PUBLIC DATABASE
PROGNOSIS	RECURRENCE	TISSUES	LOCAL COHORT
PROGNOSIS	METASTASIS	TISSUES	LOCAL COHORT
		TISSUES	PUBLIC DATABASE
		TISSUES	PUBLIC DATABASE
PROGNOSIS	METASTASIS	TISSUES	LOCAL COHORT
PROGNOSIS	GRADE	TISSUES	LOCAL COHORT
DIAGNOSIS		BLOOD	LOCAL COHORT
DIAGNOSIS		URINE	LOCAL COHORT
DIAGNOSIS		URINE	LOCAL COHORT
PROGNOSIS	METASTASIS	TISSUES	LOCAL COHORT
DIAGNOSIS		BLOOD	LOCAL COHORT
PROGNOSIS	METASTASIS	TISSUES	LOCAL COHORT
DIAGNOSIS		URINE	LOCAL COHORT
DIAGNOSIS		TISSUES	LOCAL COHORT
PROGNOSIS	PFS	TISSUES	LOCAL COHORT
DIAGNOSIS		URINE	LOCAL COHORT
PREDICTIVE	PROGRESSION	TISSUES	LOCAL COHORT
PROGNOSIS	BONE METASTASIS	TISSUES	LOCAL COHORT
PREDICTIVE	PROGRESSION	TISSUES	LOCAL COHORT
DIAGNOSIS		URINE	LOCAL COHORT
DIAGNOSIS		TISSUES	PUBLIC DATABASE
PROGNOSIS	RECURRENCE PFS	TISSUES	LOCAL COHORT/PUBI
DIAGNOSIS		URINE	LOCAL COHORT
DIAGNOSIS		TISSUES	PUBLIC DATABASE
DIAGNOSIS		TISSUES	LOCAL COHORT
DIAGNOSIS		TISSUES	LOCAL COHORT
PROGNOSIS	RECURRENCE	TISSUES	LOCAL COHORT
DIAGNOSIS		BLOOD	LOCAL COHORT
PREDICTIVE	RECURRENCE	TISSUES	LOCAL COHORT
PROGNOSIS		BLOOD	LOCAL COHORT
PROGNOSIS	OS	BLOOD	LOCAL COHORT
DIAGNOSIS		BLOOD	LOCAL COHORT
DIAGNOSIS		TISSUES	LOCAL COHORT
DIAGNOSIS		TISSUES	LOCAL COHORT
PROGNOSIS	STAGE	URINE	LOCAL COHORT
DIAGNOSIS	HIGH-RISK	URINE	LOCAL COHORT

DIAGNOSIS	HIGH-RISK	URINE	LOCAL COHORT
DIAGNOSIS		URINE	LOCAL COHORT
PROGNOSIS	OS	BLOOD	LOCAL COHORT
PROGNOSIS	METASTASIS	TISSUES	LOCAL COHORT
DIAGNOSIS		TISSUES	LOCAL COHORT
DIAGNOSIS		TISSUES	LOCAL COHORT
DIAGNOSIS		URINE	LOCAL COHORT
PROGNOSIS	RECURRENCE	TISSUES	LOCAL COHORT
PROGNOSIS	GRADE	URINE	LOCAL COHORT
PROGNOSIS	GRADE	TISSUES	LOCAL COHORT
PROGNOSIS	PFS	TISSUES	LOCAL COHORT
DIAGNOSIS		TISSUES	LOCAL COHORT
DIAGNOSIS		URINE	LOCAL COHORT
DIAGNOSIS		TISSUES	PUBLIC DATABASE
DIAGNOSIS		TISSUES	LOCAL COHORT
PROGNOSIS	OS	TISSUES	PUBLIC DATABASE
PREDICTIVE	RECURRENCE	TISSUES	PUBLIC DATABASE
PROGNOSIS	PFS	BLOOD	LOCAL COHORT
DIAGNOSIS		TISSUES	LOCAL COHORT
PROGNOSIS	PFS	TISSUES	LOCAL COHORT
PROGNOSIS	BONE METASTASIS	TISSUES	LOCAL COHORT
DIAGNOSIS		URINE	LOCAL COHORT
DIAGNOSIS		URINE	LOCAL COHORT
PROGNOSIS	RECURRENCE PFS	TISSUES	LOCAL COHORT/PUBl
DIAGNOSIS		TISSUES	LOCAL COHORT/PUB
PREDICTIVE	PROGRESSION	TISSUES	LOCAL COHORT
PREDICTIVE	PROGRESSION	TISSUES	LOCAL COHORT
DIAGNOSIS		URINE	LOCAL COHORT
DIAGNOSIS		URINE	LOCAL COHORT
DIAGNOSIS		URINE	LOCAL COHORT
DIAGNOSIS		URINE	LOCAL COHORT
DIAGNOSIS		BLOOD	LOCAL COHORT
PROGNOSIS	RECURRENCE	TISSUES	LOCAL COHORT
PROGNOSIS	RECURRENCE	TISSUES	LOCAL COHORT
DIAGNOSIS		URINE	LOCAL COHORT
DIAGNOSIS		URINE	LOCAL COHORT
DIAGNOSIS		URINE	LOCAL COHORT
DIAGNOSIS		URINE	LOCAL COHORT
DIAGNOSIS		URINE	LOCAL COHORT
DIAGNOSIS		TISSUES	LOCAL COHORT/PUB
PROGNOSIS	OS	TISSUES	LOCAL COHORT
PREDICTIVE	PROGRESSION	TISSUES	LOCAL COHORT
DIAGNOSIS	HIGH-RISK	URINE	LOCAL COHORT
DIAGNOSIS	HIGH-RISK	URINE	LOCAL COHORT
PREDICTIVE	IRAK1 therapy	TISSUES	PUBLIC DATABASE
PREDICTIVE	IRAK1 therapy	TISSUES	PUBLIC DATABASE
DIAGNOSIS		URINE	LOCAL COHORT
PROGNOSIS	METASTASIS	TISSUES	LOCAL COHORT
DIAGNOSIS		URINE	LOCAL COHORT
DIAGNOSIS		TISSUES	PUBLIC DATABASE

DIAGNOSIS		URINE	LOCAL COHORT
PREDICTIVE	RESPONSE TO TREATMENT	BLOOD	LOCAL COHORT
PROGNOSIS	RECURRENCE	TISSUES	LOCAL COHORT
PROGNOSIS	METASTASIS	TISSUES	LOCAL COHORT
DIAGNOSIS		URINE	LOCAL COHORT
PREDICTIVE	RESPONSE TO TREATMENT	BLOOD	LOCAL COHORT
PREDICTIVE	METASTASIS	tissue	LOCAL COHORT
PREDICTIVE	METASTASIS	tissue	LOCAL COHORT
PROGNOSIS	OS	TISSUES	PUBLIC DATABASE
PROGNOSIS	OS	TISSUES	PUBLIC DATABASE
DIAGNOSIS		TISSUES	PUBLIC DATABASE
PROGNOSIS	OS	TISSUES	PUBLIC DATABASE
DIAGNOSIS		BLOOD	LOCAL COHORT
PROGNOSIS	OS	TISSUES	LOCAL COHORT
DIAGNOSIS		TISSUES	LOCAL COHORT
PROGNOSIS	METASTASIS	TISSUES	LOCAL COHORT
PREDICTIVE	SURVIVAL	TISSUES	LOCAL COHORT
DIAGNOSIS		URINE	LOCAL COHORT
PREDICTIVE	RECURRENCE	TISSUES	PUBLIC DATABASE
PREDICTIVE	RECURRENCE	TISSUES	PUBLIC DATABASE
PREDICTIVE	PROGRESSION	TISSUES	LOCAL COHORT
PROGNOSIS	GRADE	TISSUES	LOCAL COHORT
PROGNOSIS	OS	TISSUES	LOCAL COHORT
DIAGNOSIS		TISSUES	PUBLIC DATABASE
		TISSUES	PUBLIC DATABASE
PREDICTIVE	RECURRENCE	TISSUES	LOCAL COHORT
DIAGNOSIS		URINE	LOCAL COHORT
DIAGNOSIS		URINE	LOCAL COHORT
DIAGNOSIS		TISSUES	LOCAL COHORT
		TISSUES	PUBLIC DATABASE
PROGNOSIS	BONE METASTASIS	TISSUES	LOCAL COHORT
PROGNOSIS	OS	TISSUES	PUBLIC DATABASE
PROGNOSIS	RECURRENCE	TISSUES	LOCAL COHORT
PROGNOSIS	STAGE	TISSUES	LOCAL COHORT
DIAGNOSIS		URINE	LOCAL COHORT
DIAGNOSIS		BLOOD	LOCAL COHORT
PROGNOSIS	STAGE	TISSUES	LOCAL COHORT
DIAGNOSIS		TISSUES	LOCAL COHORT
DIAGNOSIS		TISSUES	LOCAL COHORT
DIAGNOSIS		URINE	LOCAL COHORT
DIAGNOSIS		TISSUES	LOCAL COHORT
DIAGNOSIS		URINE	LOCAL COHORT
DIAGNOSIS		URINE	LOCAL COHORT
PROGNOSIS	STAGE	TISSUES	LOCAL COHORT
DIAGNOSIS		URINE	LOCAL COHORT
PROGNOSIS	STAGE	TISSUES	LOCAL COHORT
PROGNOSIS		TISSUES	PUBLIC DATABASE
DIAGNOSIS		URINE	LOCAL COHORT
PREDICTIVE	PROGRESSION	TISSUES	LOCAL COHORT

PROGNOSIS		TISSUES	PUBLIC DATABASE
PROGNOSIS	RECURRENCE	TISSUES	LOCAL COHORT
DIAGNOSIS			
PROGNOSIS	RECURRENCE	TISSUES	LOCAL COHORT
PREDICTIVE	RECURRENCE	TISSUES	PUBLIC DATABASE
PREDICTIVE	PROGRESSION	TISSUES	LOCAL COHORT
PREDICTIVE	PROGRESSION	TISSUES	LOCAL COHORT
DIAGNOSIS		TISSUES	LOCAL COHORT
PROGNOSIS	GRADE	TISSUES	LOCAL COHORT
DIAGNOSIS		URINE	LOCAL COHORT
PROGNOSIS		TISSUES	PUBLIC DATABASE
DIAGNOSIS		URINE	LOCAL COHORT
DIAGNOSIS		BLOOD	LOCAL COHORT
PROGNOSIS	GRADE	TISSUES	LOCAL COHORT
PROGNOSIS	METASTASIS	TISSUES	LOCAL COHORT
PROGNOSIS	METASTASIS	TISSUES	LOCAL COHORT
PREDICTIVE	METASTASIS	tissue	LOCAL COHORT
PREDICTIVE	SURVIVAL	TISSUES	LOCAL COHORT
PROGNOSIS	INVASION	TISSUES	LOCAL COHORT
PREDICTIVE	SURVIVAL	TISSUES	LOCAL COHORT
PROGNOSIS	GRADE PFS	TISSUES	LOCAL COHORT
PROGNOSIS	RECURRENCE PFS	TISSUES	LOCAL COHORT
PROGNOSIS	METASTASIS	BLOOD	LOCAL COHORT
DIAGNOSIS		TISSUES	PUBLIC DATABASE
PREDICTIVE	METASTASIS	BLOOD	LOCAL COHORT
PROGNOSIS	METASTASIS	TISSUES	LOCAL COHORT
PREDICTIVE	PROGRESSION	TISSUES	LOCAL COHORT
PROGNOSIS	PFS	TISSUES	LOCAL COHORT/PUB
DIAGNOSIS		TISSUES	LOCAL COHORT/PUB
PREDICTIVE	RECURRENCE	TISSUES	PUBLIC DATABASE
pROGNOSIS	RECURRENCE GRADE	TISSUES	PUBLIC DATABASE
PREDICTIVE	METASTASIS	tissue	LOCAL COHORT
PROGNOSIS	RECURRENCE	TISSUES	LOCAL COHORT
PROGNOSIS	METASTASIS	TISSUES	LOCAL COHORT
PROGNOSIS	PFS	TISSUES	LOCAL COHORT
PROGNOSIS	STAGE	TISSUES	LOCAL COHORT
PROGNOSIS	OS	TISSUES	LOCAL COHORT
PROGNOSIS	OS	TISSUES	LOCAL COHORT/PUB
DIAGNOSIS		URINE	LOCAL COHORT
DIAGNOSIS		URINE	LOCAL COHORT
DIAGNOSIS		TISSUES	LOCAL COHORT
PREDICTIVE	PROGRESSION	TISSUES	LOCAL COHORT
DIAGNOSIS		TISSUES	PUBLIC DATABASE
PROGNOSIS		TISSUES	PUBLIC DATABASE
PREDICTIVE	METASTASIS	tissue	LOCAL COHORT
PREDICTIVE	RECURRENCE	TISSUES	LOCAL COHORT
PREDICTIVE	RECURRENCE	TISSUES	PUBLIC DATABASE
		TISSUES	PUBLIC DATABASE
DIAGNOSIS		TISSUES	LOCAL COHORT
DIAGNOSIS		URINE	

DIAGNOSIS		BLOOD	LOCAL COHORT
DIAGNOSIS		TISSUES	PUBLIC DATABASE
DIAGNOSIS		TISSUES	PUBLIC DATABASE
DIAGNOSIS		BLOOD	LOCAL COHORT
PROGNOSIS	PFS	TISSUES	LOCAL COHORT
PROGNOSIS	RECURRENCE	BLOOD	LOCAL COHORT
DIAGNOSIS	HIGH-RISK	URINE	LOCAL COHORT
DIAGNOSIS		TISSUES	LOCAL COHORT
PREDICTIVE	PROGRESSION	TISSUES	LOCAL COHORT
PROGNOSIS	OS	TISSUES	PUBLIC DATABASE
DIAGNOSIS		TISSUES	PUBLIC DATABASE
PROGNOSIS	METASTASIS	TISSUES	LOCAL COHORT
PROGNOSIS	METASTASIS	TISSUES	LOCAL COHORT
PROGNOSIS	STAGE	URINE	LOCAL COHORT
PROGNOSIS	RECURRENCE	TISSUES	LOCAL COHORT
DIAGNOSIS		TISSUES	LOCAL COHORT
PROGNOSIS	METASTASIS	TISSUES	LOCAL COHORT
DIAGNOSIS		TISSUES	LOCAL COHORT
DIAGNOSIS		URINE	LOCAL COHORT
DIAGNOSIS		URINE	LOCAL COHORT
DIAGNOSIS		BLOOD	LOCAL COHORT
DIAGNOSIS		TISSUES	LOCAL COHORT
DIAGNOSIS		TISSUES	PUBLIC DATABASE
PROGNOSIS	GRADE	TISSUES	LOCAL COHORT
DIAGNOSIS		TISSUES	LOCAL COHORT
DIAGNOSIS		TISSUES	LOCAL COHORT
PROGNOSIS	OS	BLOOD	LOCAL COHORT
DIAGNOSIS		BLOOD	LOCAL COHORT
PROGNOSIS	STAGE	URINE	LOCAL COHORT
DIAGNOSIS		URINE	LOCAL COHORT
PROGNOSIS	METASTASIS	TISSUES	LOCAL COHORT
		BLOOD	LOCAL COHORT
DIAGNOSIS		BLOOD	LOCAL COHORT
DIAGNOSIS		URINE	LOCAL COHORT
PREDICTIVE	METASTASIS	tissue	LOCAL COHORT
PREDICTIVE	PROGRESSION	TISSUES	LOCAL COHORT
PROGNOSIS	PFS	TISSUES	LOCAL COHORT
DIAGNOSIS		TISSUES	LOCAL COHORT
PROGNOSIS	METASTASIS	TISSUES	LOCAL COHORT
PROGNOSIS	OS	TISSUES	PUBLIC DATABASE
PREDICTIVE	METASTASIS	tissue	LOCAL COHORT
PREDICTIVE	METASTASIS	tissue	LOCAL COHORT
PROGNOSIS	OS	TISSUES	PUBLIC DATABASE
DIAGNOSIS		TISSUES	PUBLIC DATABASE
PROGNOSIS	OS	TISSUES	PUBLIC DATABASE
PROGNOSIS	METASTASIS	TISSUES	LOCAL COHORT
DIAGNOSIS		TISSUES	PUBLIC DATABASE
PROGNOSIS	METASTASIS	TISSUES	LOCAL COHORT
DIAGNOSIS		TISSUES	PUBLIC DATABASE

PROGNOSIS	GRADE RECURRENCE	TISSUES	LOCAL COHORT
DIAGNOSIS		TISSUES	LOCAL COHORT
DIAGNOSIS		TISSUES	LOCAL COHORT/PUBI
PREDICTIVE	PROGRESSION	TISSUES	LOCAL COHORT
DIAGNOSIS		BLOOD	LOCAL COHORT
DIAGNOSIS		URINE	LOCAL COHORT
PROGNOSIS		TISSUES	PUBLIC DATABASE
DIAGNOSIS		URINE	LOCAL COHORT
DIAGNOSIS		URINE	LOCAL COHORT
DIAGNOSIS	HIGH-RISK	URINE	LOCAL COHORT
DIAGNOSIS		TISSUES	LOCAL COHORT
PROGNOSIS	PFS	TISSUES	PUBLIC DATABASE
DIAGNOSIS		TISSUES	PUBLIC DATABASE
PROGNOSIS	METASTASIS	TISSUES	LOCAL COHORT
PROGNOSIS	METASTASIS	TISSUES	LOCAL COHORT
PROGNOSIS	STAGE RECURRENCE	TISSUES	LOCAL COHORT
PREDICTIVE	PROGRESSION	TISSUES	LOCAL COHORT
PROGNOSIS	METASTASIS	TISSUES	LOCAL COHORT
PROGNOSIS	RECURRENCE	TISSUES	PUBLIC DATABASE
PROGNOSIS	OS	BLOOD	LOCAL COHORT
		TISSUES	PUBLIC DATABASE
DIAGNOSIS		TISSUES	PUBLIC DATABASE
DIAGNOSIS		TISSUES	PUBLIC DATABASE
DIAGNOSIS		TISSUES	PUBLIC DATABASE
DIAGNOSIS		TISSUES	PUBLIC DATABASE
DIAGNOSIS	GRADE	TISSUES	LOCAL COHORT
PROGNOSIS	GRADE	TISSUES	LOCAL COHORT
DIAGNOSIS		TISSUES	PUBLIC DATABASE
DIAGNOSIS		BLOOD	LOCAL COHORT
DIAGNOSIS		URINE	LOCAL COHORT
DIAGNOSIS		BLOOD	LOCAL COHORT
DIAGNOSIS		TISSUES	LOCAL COHORT
DIAGNOSIS		BLOOD	LOCAL COHORT
PROGNOSIS	METASTASIS	TISSUES	LOCAL COHORT
PROGNOSIS	METASTASIS	TISSUES	LOCAL COHORT
PROGNOSIS	OS	TISSUES	PUBLIC DATABASE
PROGNOSIS	RECURRENCE	BLOOD	LOCAL COHORT
PROGNOSIS	METASTASIS	TISSUES	LOCAL COHORT
DIAGNOSIS		URINE	LOCAL COHORT
DIAGNOSIS		TISSUES	LOCAL COHORT
DIAGNOSIS		TISSUES	PUBLIC DATABASE
DIAGNOSIS		TISSUES	LOCAL COHORT
DIAGNOSIS		TISSUES	LOCAL COHORT
PREDICTIVE	SURVIVAL	TISSUES	LOCAL COHORT
DIAGNOSIS		TISSUES	LOCAL COHORT
PROGNOSIS	PFS	TISSUES	LOCAL COHORT
DIAGNOSIS		TISSUES	LOCAL COHORT
PROGNOSIS	RECURRENCE	TISSUES	LOCAL COHORT
PROGNOSIS	RECURRENCE	TISSUES	LOCAL COHORT
PROGNOSIS	RECURRENCE PFS	TISSUES	LOCAL COHORT

PROGNOSIS	OS	TISSUES	LOCAL COHORT
DIAGNOSIS		BLOOD	LOCAL COHORT
DIAGNOSIS		TISSUES	LOCAL COHORT
PROGNOSIS		BLOOD	LOCAL COHORT
PROGNOSIS	GRADE	TISSUES	LOCAL COHORT
PROGNOSIS	METASTASIS	TISSUES	LOCAL COHORT
PROGNOSIS	METASTASIS	TISSUES	LOCAL COHORT
DIAGNOSIS		TISSUES	PUBLIC DATABASE
PROGNOSIS	RECURRENCE PFS	TISSUES	LOCAL COHORT/PUB
PREDICTIVE	PROGRESSION	TISSUES	LOCAL COHORT
PROGNOSIS	GRADE	TISSUES	LOCAL COHORT
PREDICTIVE	METASTASIS	tissue	LOCAL COHORT
PROGNOSIS	BONE METASTASIS	TISSUES	LOCAL COHORT
DIAGNOSIS		TISSUES	LOCAL COHORT
PROGNOSIS		BLOOD	LOCAL COHORT
PROGNOSIS	METASTASIS	TISSUES	LOCAL COHORT
		TISSUES	PUBLIC DATABASE
DIAGNOSIS		URINE	LOCAL COHORT
PREDICTIVE	PROGRESSION	TISSUES	LOCAL COHORT
PREDICTIVE	PROGRESSION	TISSUES	LOCAL COHORT
PREDICTIVE	PROGRESSION	TISSUES	LOCAL COHORT
DIAGNOSIS		URINE	LOCAL COHORT
PROGNOSIS	METASTASIS	TISSUES	LOCAL COHORT
DIAGNOSIS		URINE	LOCAL COHORT
PROGNOSIS	OS	TISSUES	PUBLIC DATABASE
PROGNOSIS	GRADE	TISSUES	LOCAL COHORT
DIAGNOSIS		URINE	LOCAL COHORT
PROGNOSIS	RECURRENCE	TISSUES	PUBLIC DATABASE
PREDICTIVE	RESISTANCE	TISSUES	LOCAL COHORT
PREDICTIVE	METASTASIS	tissue	LOCAL COHORT
PREDICTIVE	PROGRESSION	TISSUES	LOCAL COHORT
PREDICTIVE	RECURRENCE	TISSUES	LOCAL COHORT
DIAGNOSIS		URINE	LOCAL COHORT
PROGNOSIS	RECURRENCE	TISSUES	LOCAL COHORT
PREDICTIVE	RECURRENCE	TISSUES	PUBLIC DATABASE
PREDICTIVE	RECURRENCE	TISSUES	PUBLIC DATABASE
PREDICTIVE	RECURRENCE	TISSUES	PUBLIC DATABASE
PREDICTIVE	RECURRENCE	TISSUES	PUBLIC DATABASE
PREDICTIVE	RECURRENCE	TISSUES	PUBLIC DATABASE
PREDICTIVE	RECURRENCE	TISSUES	PUBLIC DATABASE
DIAGNOSIS		TISSUES	
DIAGNOSIS		TISSUES	
DIAGNOSIS		TISSUES	
DIAGNOSIS		TISSUES	
DIAGNOSIS		TISSUES	
DIAGNOSIS		TISSUES	
DIAGNOSIS		TISSUES	
DIAGNOSIS		TISSUES	
DIAGNOSIS		TISSUES	
DIAGNOSIS		TISSUES	

DIAGNOSIS		TISSUES	
DIAGNOSIS		TISSUES	
DIAGNOSIS		TISSUES	
DIAGNOSIS		TISSUES	
DIAGNOSIS		TISSUES	
DIAGNOSIS		TISSUES	LOCAL COHORT/PUB

SPECIFIC CG	REFERENCE	
cg14034476	Cheng et al 2021	
cg06942685	Cheng et al 2021	
chr2:27958210-27958689	Pidsley et al 2022	
cg21438754	Chao et al 2023	
cg24718437	Chao et al 2023	
cg22408108	Cheng et al 2021	
cg23883265	Chao et al 2023	
cg05085627	Chao et al 2023	
	Daniunaite et al 2021	
cg15002904	Ruiz-Deya et al 2021	
	Brikun et al 2018	
cg10586836	Chao et al 2023	
	Habeshian et al 2024	
	Habeshian et al 2024	
cg17327934	Chao et al 2023	
	Dillinger et al 2022	
	Xu n et al 2019	
cg08679466	Chao et al 2023	
cg07166550	Zhao S et al 2017	
cg07166550	Zhao S et al 2017	
	Angulo et al 2016	
	Tong Y et al 2019	
cg22953017, Cg13875120, cg12627583, cg04380340	Møller et al 2017	
cg08952506	Geybels et al 2015	
	Geybels et al 2015b	
	Brikun et al 2018	
cg06558491	Chao et al 2023	
	SUP	
	Jeyapal et al 2020	
	Patel et al 2019	
	O'Reilly et al 2019	
	Moreira-Barbosa et al 2018	
	Hendriks et al 2018	
	Van Neste et al 2016	
	Rybicki et al 2016	
	atkoe et al 2015	
	Eismann et al 2023	
	Litovkin et al 2015	
	Zhang W et al 2023	
	Brikun et al 2018	
	Nekrasov et al 2019	
	Ylitalo et al 2021	
	Bernatz et al 2024	
	FitzGerald et al 2024	
CG02210149	Liu et al 2022	
cg21513610	Zhao S et al 2017	
	Wang L et al 2020	
	FitzGerald et al 2024	
chrX:102000717-1020015	Pidsley et al 2022	

cg19500311	Tonmoy et al 2022	
cg23614229	Tonmoy et al 2022	
chr12:1906206-1906676	Pidsley et al 2022	
chr14:90849492-90850589	Pidsley et al 2022	
CG14477205 to CG24294857	Peng et al 2023	
	Wang L et al 2020	
	Yang B et al 2019	
	Skara et al 2023	
cg23824801	Tang Y et al 2017	
	Patel et al 2019	
cg03576469	Tang Y et al 2017	
	Luo et al 2022	
	Habeshian et al 2024	
	Gurioli et al 2016	
	Ahmad et al 2016	
	Litovkin et al 2015	
	Zhang W et al 2023	
chr1:208132439-208132824	Pidsley et al 2022	
	Luo et al 2022	
	Wilkinson et al 2024	
	Habeshian et al 2024	
	Nekrasov et al 2019	
	Moses-Fynn et al 2018	
chr8:95246476-95246871	Pidsley et al 2022	
chr5:115151283-115152645	Pidsley et al 2022	
LIC DATABASE	Meller et al 2016	
CG18786969	Liu et al 2022	
cg21472468	Chao et al 2023	
cg17113117	Chao et al 2023	
cg16926102	Liu B et al 2019	
cg19710323	Liu B et al 2019	
cg11176151	Chao et al 2023	
cg01232206	Chao et al 2023	
cg26160150	Chao et al 2023	
cg25422753	Chao et al 2023	
cg04278212	Chao et al 2023	
cg07333191	Liu B et al 2019	
cg22620090	Liu B et al 2019	
cg16389386	Liu B et al 2019	
cg10232918	Chao et al 2023	
cg18209323	Chao et al 2023	

cg17811434	Chao et al 2023	
	Dillinger et al 2022	
	Dillinger et al 2022	
	Sha P et al 2024	
	FitzGerald et al 2024	
chr13:53312994-53313591	Pidsley et al 2022	
	Xu n et al 2019	
	Strand et al 2017	
cg17713488	Ruiz-Deya et al 2021	
g22030684	Ruiz-Deya et al 2021	
	Habeshian et al 2024	
	Dillinger et al 2022	
chr12:3862069-3862497	Pidsley et al 2022	
	Wang L et al 2020	
	SAVIO et al 2020	
	Jeyapal et al 2020	
	Qin et al 2022	
	Huang Q-X et al 2018	
	Gao X et al 2017	
cg23679434	Cheng et al 2021	
cg02893550	Cheng et al 2021	
	Dillinger et al 2022	
LIC DATABASE	Goltz et al 2016	
	Brikun et al 2018	
	Moses-Fynn et al 2018	
cg08843517	Tang Y et al 2017	
	Horning et al 2015	
	Luo et al 2022	
	FitzGerald et al 2024	
cg22669123	Ruiz-Deya et al 2021	
	LAN et al 2021	
	Tong Y et al 2019	
	Lee J et al 2016	
chr11:93063135-93064069	Pidsley et al 2022	
	Bjerre et al 2020	
LIC DATABASE	Bjerre et al 2019	
	Ahmad et al 2016	
	Moses-Fynn et al 2018	
	FitzGerald et al 2024	
CG23309257	Liu et al 2022	
cg05385513	Tang Y et al 2017	
	FitzGerald et al 2024	
CG00210002	Liu et al 2022	
	Luo et al 2022	
cg02488349	Chao et al 2023	
	Liao Y et al 2022	
	Li S et al 2015	
chr3:184243657-184243936	Pidsley et al 2022	

	Brikun et al 2018	
	Angulo et al 2016b	
	Yang B et al 2019	
cg01397449	Liu B et al 2019	
cg23957912	Tonmoy et al 2022	
cg04992588 cg24659328 cg2615468 cg01446993	Ke et al 2022	
cg25161377	Ruiz-Deya et al 2021	
cg10206627	Chao et al 2023	
CG11314056	Liu et al 2022	
CG22011804	Liu et al 2022	
	Tong Y et al 2019	
cg23095612	Bjerre et al 2020	
	Yang B et al 2019	
cg06313119	Cheng et al 2021	
cg02394978	Zhao S et al 2017	
cg02394978	Zhao S et al 2017	
	Wang L et al 2020	
	Wang L et al 2020	
	Rubicz et al 2019	
	Wu Y et al 2016	
	Constâncio et al 2019	
	Constancio et al 2019	
	Nekrasov et al 2019	
cg14539730	Ruiz-Deya et al 2021	
	Reis et al 2015	
cg11747142	Ruiz-Deya et al 2021	
	Sha P et al 2024	
	Patel et al 2019	
	Ashour et al 2020	
	Brikun et al 2018	
CG08612779	Liu et al 2022	
	Ylitalo et al 2021	
CG26862690	Liu et al 2022	
	Brikun et al 2019	
	Xu n et al 2019	
LIC DATABASE	Bjerre et al 2019	
	Sha P et al 2024	
	Xu n et al 2019	
	Patel et al 2019	
	FitzGerald et al 2024	
	Angulo et al 2016	
	Bryzgunova et al 2021	
	Witt et al 2022	
	Friedemann et al 2021	
	Zavridou et al 2021	
	Constâncio et al 2019	
	FIANO et al 2019	
	Patel et al 2019	
	Bakavicius et al 2019	
	O'Reilly et al 2019	

	Zhao F et al 2018	
	Brikun et al 2018	
	Hendriks et al 2018	
cg22224704, cg06928838, cg02659086	Møller et al 2017	
	Martignano et al 2016	
	Van Neste et al 2016	
	Minciu et al 2016	
	Litovkin et al 2015	
	atkoe et al 2015	
	Eismann et al 2023	
	Pidsley et al 2024	
	Gurioli et al 2016	
	Moreira-Barbosa et al 2018	
	Zhang W et al 2023	
	Gurioli et al 2016	
cg03482458	Cheng et al 2021	
	Luo et al 2022	
	Bjerre et al 2020	
	Patel et al 2019	
cg04829853, cg03628719	Møller et al 2017	
	Ylitalo et al 2021	
	Brikun et al 2018	
	Sha P et al 2024	
LIC DATABASE	Bjerre et al 2019	
cg14117138	Geybels et al 2015	
CG14024461	Liu et al 2022	
CG17177673	Liu et al 2022	
	Brikun et al 2019	
	Brikun et al 2018	
	Brikun et al 2018	
	Brikun et al 2018	
	Constâncio et al 2019	
	SAVIO et al 2020	
	Jeyapal et al 2020	
	Brikun et al 2019	
	Zhao F et al 2018	
	Brikun et al 2018	
	Brikun et al 2018	
	Brikun et al 2018	
cg16508627	Liu B et al 2019	
	Ahmad et al 2016	
CG02593924	Liu et al 2022	
	O'Reilly et al 2019	
	O'Reilly et al 2019	
cg23604959	Schagdarsurengin et al 2022	
cg02742918	Schagdarsurengin et al 2022	
	Sha P et al 2024	
cg17983571	Ruiz-Deya et al 2021	
	Sha P et al 2024	
cg20883831	Tang Y et al 2017	

	Brikun et al 2018	
	Dillinger et al 2022	
cg16713292	Zhao S et al 2017	
cg16713292	Zhao S et al 2017	
	Brikun et al 2019	
	Dillinger et al 2022	
cg07280593	Chao et al 2023	
cg06789519	Chao et al 2023	
cg15736169	Cheng et al 2021	
cg21741562	Cheng et al 2021	
	Nikas & Nikas 2019	
cg09671962	Cheng et al 2021	
	Moses-Fynn et al 2018	
	Fiano et al 2017	
	FitzGerald et al 2024	
	Rubicz et al 2019	
chr17:62773682-62777796	Pidsley et al 2022	
	Nekrasov et al 2019	
	Luo et al 2022	
	Luo et al 2022	
CG12195149	Liu et al 2022	
	FitzGerald et al 2024	
	Ahmad et al 2016	
	Xu n et al 2019	
	Wang L et al 2020	
chr5:10333634-10334055	Pidsley et al 2022	
	Sha P et al 2024	
	Sha P et al 2024	
	FitzGerald et al 2024	
	Wang L et al 2020	
	Ylitalo et al 2021	
cg14245102	Cheng et al 2021	
cg06933370 cg25181383	NORGAARD et al 2019	
cg05850656	Torres-Ferreira et al 2017	
cg05850656	Torres-Ferreira et al 2017	
	Friedemann et al 2024	
cg14416371	Torres-Ferreira et al 2017	
cg05687686	Torres-Ferreira et al 2017	
	Kaukoniemi et al 2015	
	Moreira-Barbosa et al 2018	
cg09918657	Torres-Ferreira et al 2017	
cg09918657	Torres-Ferreira et al 2017	
	Moreira-Barbosa et al 2018	
cg22879515	Torres-Ferreira et al 2017	
cg22879515	Torres-Ferreira et al 2017	
cg08304190	Torres-Ferreira et al 2017	
cg00576773	Tonmoy et al 2022	
	Brikun et al 2018	
CG08917831	Liu et al 2022	

cg11052780	Tonmoy et al 2022	
	Angulo et al 2016	
	Junjie Chen 2020	
	Angulo et al 2016	
	Daniunaite et al 2021	
CG10388316	Liu et al 2022	
CG024554483	Liu et al 2022	
	Yang B et al 2019	
	FitzGerald et al 2024	
	Brikun et al 2018	
cg12626968	Tonmoy et al 2022	
	Brikun et al 2018	
	Friedemann et al 2024	
	Wu Y et al 2016	
cg21359838	Ruiz-Deya et al 2021	
	Habeshian et al 2024	
cg09560488	Chao et al 2023	
chr12:103311054-103311276	Pidsley et al 2022	
	Lounglaithong et al 2018	
chr15:72564636-72565252	Pidsley et al 2022	
	Deng Q-K et al 2016	
	Lin Y-L et al 2015	
	Lin Y et al 2017	
cg06575035	Liu B et al 2019	
	Dillinger et al 2022	
cg25641223	Ruiz-Deya et al 2021	
CG04351156	Liu et al 2022	
cg19724470	Gevensleben et al 2016	
LIC DATABASEPUBLIC DATABASE	Bjerre et al 2019	
	Sun et al 2022	
	Sun J et al 2022	
cg26341465	Chao et al 2023	
cg24349665	Zhao S et al 2017	
cg24349665	Zhao S et al 2017	
	Uhl et al 2017	
	Luan Z et al 2016	
	Ahmad et al 2016	
cg12324970, cg23095743	Holmes et al 2016	
	Khemees et al 2021	
	Jarrard et al 2019	
	Yang B et al 2019	
CG01188584	Liu et al 2022	
	Tong Y et al 2019	
cg05850997	Tonmoy et al 2022	
cg03347306	Chao et al 2023	
chr4:81118427-81118588	Pidsley et al 2022	
	Daniunaite et al 2021	
	Wang L et al 2020	
	FitzGerald et al 2024	
cg05163709	Yao L et al 2015	

	Wang Y et al 2024	
	Zhang W et al 2023	
	Zheng Dai et al 2024	
	Zheng Dai et al 2024	
	Strand et al 2017	
	Dillinger et al 2022	
	O'Reilly et al 2019	
	Litovkin et al 2015	
CG18976893	Liu et al 2022	
cg24514600	Cheng et al 2021	
	Tong Y et al 2019	
cg09154639	Ruiz-Deya et al 2021	
	Habeshian et al 2024	
	Bakavicius et al 2019	
	Angulo et al 2016	
	Litovkin et al 2015	
	Habeshian et al 2024	
	Gurioli et al 2016	
	Constancio et al 2019	
	Moreira-Barbosa et al 2018	
	Moses-Fynn et al 2018	
	Patel et al 2019	
	Zhang W et al 2023	
	Eismann et al 2023	
	Gurioli et al 2016	
	Van Neste et al 2016	
	Zavridou et al 2021	
	Constâncio et al 2019	
	Bakavicius et al 2019	
	Constancio et al 2019	
	Habeshian et al 2024	
	Friedemann et al 2021	
	Friedemann et al 2024	
	Brikun et al 2018	
cg15341132	Chao et al 2023	
CG17400113	Liu et al 2022	
	Strand et al 2017	
	Geybels et al 2015b	
cg25921194	Ruiz-Deya et al 2021	
cg06457534	Cheng et al 2021	
cg16030580	Chao et al 2023	
cg09104747	Chao et al 2023	
cg23643814	Cheng et al 2021	
cg26733975	Liu B et al 2019	
cg00496102	Cheng et al 2021	
	Bernatz et al 2024	
	Nikas & Nikas 2019	
cg15218485	Ruiz-Deya et al 2021	
	Xu n et al 2019	

	Eismann et al 2023	
	Gurioli et al 2016	
LIC DATABASELOCAL COHORT	Geybels et al 2015	
CG13834623	Liu et al 2022	
	Moses-Fynn et al 2018	
	Sha P et al 2024	
cg23194354	Tonmoy et al 2022	
	Sha P et al 2024	
	Constancio et al 2019	
	O'Reilly et al 2019	
	FitzGerald et al 2024	
	Ma J et al 2024)	
cg06594281, cg04305621, cg10829727, cg09300114, cg045[illegible]	Meng et al 2021	
cg24163360	Ruiz-Deya et al 2021	
cg00498305, cg19617377	Møller et al 2017	
	Haldrup et al 2016	
CG16129800	Liu et al 2022	
	Rubicz et al 2019	
cg06480736, cg19774478, cg19788741	Li X et al 2019	
	Zavridou et al 2021	
	Wang L et al 2020	
	Tong Y et al 2019	
cg26642667	Liu B et al 2019	
	Nikas & Nikas 2019	
cg06363129, cg07220448, cg11417025	Tang Y et al 2017	
	Pugongchai et al 2017	
	Pugonchai et al 2017	
cg04374393	Liu B et al 2019	
	Constâncio et al 2019	
	Constancio et al 2019	
	Friedemann et al 2024	
	Yang B et al 2019	
	Moses-Fynn et al 2018	
	Liu T et al 2017	
cg19092163	Ruiz-Deya et al 2021	
	Wang Z et al 2020	
	Horning et al 2015	
	Wilkinson et al 2024	
	Sha P et al 2024	
	Haldrup et al 2018	
cg15089950	Rocha et al 2021	
	FitzGerald et al 2024	
	FitzGerald et al 2024	
chr22:19742681-19743728	Pidsley et al 2022	
	FitzGerald et al 2024	
	Strand et al 2017	
	FitzGerald et al 2024	
	SAVIO et al 2020	
	Jeyapal et al 2020	
CG11625005	Pedro Castelo-Branco 2016	

	Ahmad et al 2016	
	Moses-Fynn et al 2018	
	Geybels et al 2015b	
	Dillinger et al 2022	
	Eismann et al 2023	
cg05258834	Ruiz-Deya et al 2021	
	Rubicz et al 2019	
cg17804348	Liu B et al 2019	
LIC DATABASEPUBLIC DATABASE	Bjerre et al 2019	
CG23088461	Liu et al 2022	
	FitzGerald et al 2024	
cg25661986	Chao et al 2023	
	Ylitalo et al 2021	
	FitzGerald et al 2024	
cg23982858, cg009275554, cg22538054, and cg03308628	Londra et al 2021	
	Habeshian et al 2024	
	Wang L et al 2020	
	Khemees et al 2021	
CG1876823	Liu et al 2022	
CG27141120	Liu et al 2022	
CG01101400	Liu et al 2022	
	Sha P et al 2024	
	Wilkinson et al 2024	
	Nekrasov et al 2019	
cg19930288	Cheng et al 2021	
	FitzGerald et al 2024	
	Nekrasov et al 2019	
cg03234186, cg12506930, cg2646539	Zhang W et al 2018	
	Angulo et al 2016b	
cg22356268	Chao et al 2023	
CG14603375	Liu et al 2022	
chr7:99155673-99157071	Pidsley et al 2022	
	Sha P et al 2024	
	Haldrup et al 2018	
cg01223512	Zhu Jet al 2022	
cg05241265	Zhu Jet al 2022	
cg09129050	Zhu Jet al 2022	
cg16046505	Zhu Jet al 2022	
cg24250070	Zhu Jet al 2022	
cg26108999	Zhu Jet al 2022	
g18800143	Lang Wu et al 2020	
cg07645299	Lang Wu et al 2020	
cg12627844	Lang Wu et al 2020	
cg16397176	Lang Wu et al 2020	
cg11562153	Lang Wu et al 2020	
cg13866093	Lang Wu et al 2020	
cg24388424	Lang Wu et al 2020	
cg00444740	Lang Wu et al 2020	
cg06836406	Lang Wu et al 2020	
cg20100049	Lang Wu et al 2020	

cg22370235	Lang Wu et al 2020	
cg04739953	Lang Wu et al 2020	
cg01715842	Lang Wu et al 2020	
cg13230424	Lang Wu et al 2020	
cg23397578	Lang Wu et al 2020	
cg12799885	Bjerre et al 2019	
